# Dispersion-engineered spin photonics based on folded-path metasurfaces

**DOI:** 10.1038/s41377-025-01850-w

**Published:** 2025-05-16

**Authors:** Fei Zhang, Hanlin Bao, Mingbo Pu, Yinghui Guo, Tongtong Kang, Xiong Li, Qiong He, Mingfeng Xu, Xiaoliang Ma, Xiangang Luo

**Affiliations:** 1 National Key Laboratory of Optical Field Manipulation Science and Technology, Institute of Optics and Electronics, Chinese Academy of Sciences, Chengdu, 610209 China; 2 State Key Laboratory of Optical Technologies on Nano-Fabrication and Micro-Engineering, Institute of Optics and Electronics, Chinese Academy of Sciences, Chengdu, 610209 China; 3 College of Materials Sciences and Opto-Electronic Technology, University of Chinese Academy of Sciences, Beijing, 100049 China; 4 Research Center on Vector Optical Fields, Institute of Optics and Electronics, Chinese Academy of Sciences, Chengdu, 610209 China

**Keywords:** Metamaterials, Sub-wavelength optics

## Abstract

Spin photonics revolutionizes photonic technology by enabling precise manipulation of photon spin states, with spin-decoupled metasurfaces emerging as pivotal in complex optical field manipulation. Here, we propose a folded-path metasurface concept that enables independent dispersion and phase control of two opposite spin states, effectively overcoming the limitations of spin photonics in achieving broadband decoupling and higher integration levels. This advanced dispersion engineering is achieved by modifying the equivalent length of a folded path, generated by a virtual reflective surface, in contrast to previous methods that depended on effective refractive index control by altering structural geometries. Our approach unlocks previously unattainable capabilities, such as achieving achromatic focusing and achromatic spin Hall effect using the rotational degree of freedom, and generating spatiotemporal vector optical fields with only a single metasurface. This advancement substantially broadens the potential of metasurface-based spin photonics, extending its applications from the spatial domain to the spatiotemporal domain.

## Abstract (web summary)

A folded-path metasurface concept is proposed to simultaneously achieve independent dispersion control and wavefront shaping for arbitrary orthogonal polarizations.

## Graphical abstract

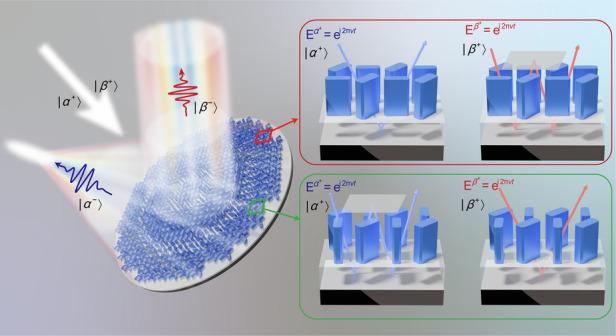

## Introduction

Spin photonics is an innovative field that merges the concepts of spintronics and photonics, leveraging the spin and polarization properties of photons for advanced information processing and transmission^1,2^. When the spin angular momentum of photons interacts with their orbital angular momentum, it can give rise to anomalous phenomena and novel applications, such as the well-known geometric phase and photonic spin Hall effect (PSHE)^3–7^. In recent years, some important progress has been made in the field of spin photonics, such as optical precision measurement^8,9^, differential operation and imaging^10,11^, and high-contrast microscopy^12,13^, multi-dimensional information multiplexing^14^. However, spin–orbit interactions typically exhibit strong conjugate symmetry, which limits the information multiplexing capability in two spin states^15–17^. This symmetry can be disrupted by integrating spin-dependent geometric phases with spin-independent propagation phases, thereby enabling asymmetric spin–orbit interactions^18–20^. It has found extensive applications in many areas, such as three-dimensional holography^21^, optical field manipulation^22–24^, polarization imaging^25,26^, quantum optics^27,28^, programmable optical platform^29,30^, and many others^31,32^. However, previous spin-decoupled metasurfaces have mainly focused on single-wavelength or narrow-band applications, failing to achieve broadband spin decoupling due to a lack of dispersion control. Optical dispersion occurs due to variations in a material’s refractive index with the frequency of the incident light. Unlike traditional refractive optics, the effective refractive index and dispersion of optical metasurfaces composed of subwavelength nanostructures are determined mainly by the arrangement and geometry of these structures, rather than by their material composition. This characteristic results in an excellent ability to accurately control dispersion. Dispersion-engineered metasurfaces have garnered significant attention and have been widely employed to realize broadband achromatic focusing and imaging^33–40^, augmented and virtual reality displays^41,42^, spectral detection and imaging^43–45^, spatiotemporal optical field control^46–48^, among many others^49,50^.

In general, approaches for achieving dispersion control can be broadly categorized into two types^51–53^. The first involves increasing the height of nanostructures or employing multi-layer structures^39–41,54,55^. The second relies on structures with intricate cross-sections to achieve dispersion control^33–37,50^. These complex structural geometries often complicate the design, simulation, and fabrication processes^56^. More importantly, existing dispersion engineering methods are either spin-independent or applicable only to a single spin state, due to the fact that the dispersive propagation phase is spin-independent, while the dispersion-free geometric phase is spin-conjugate^18,57^. Chiral meta-atoms were proposed to break spin-conjugate in geometric phase with robust and broadband properties, enriching the design of metasurfaces^58^. The broadband performance results in the dispersion-free property, lacking versatility in independent dispersion control for arbitrary two orthogonal polarizations. The development of spin photonics toward broadband decoupling and higher integration levels has faced significant limitations due to the lack of independent dispersion control for opposite spin states at subwavelength scales. For example, spatiotemporal vector optical field manipulation typically requires two separate Fourier transform setups to independently control the phase and dispersion of two orthogonal polarization components^47,48,59^, resulting in a cumbersome and bulky system.

Here, we present a concept of folded-path metasurfaces to achieve spin-decoupled dispersion control. The achievement of such an unprecedented ability does not require complex structural design. The key idea is to modify the equivalent path length by folding the light path through local interference at subwavelength scales. By carefully engineering polarization-decoupled interference, it is demonstrated to achieve independent dispersion control and versatile wavefront shaping for any pair of orthogonal states of polarization, whether they are linear, circular, or elliptical. We showcase several capabilities that were previously unattainable with traditional designs, such as the realization of achromatic focusing and achromatic PSHE using rotational degrees of freedom, and the generation of spatiotemporal vector optical fields with a single metasurface. This metasurface platform is expected to unlock possibilities for compact spin-multiplexing devices for various applications, such as broadband polarization optics, information encoding, and spatiotemporal optical field manipulation.

## Results

### Principle of folded-path metasurfaces

**Fig. 1:**
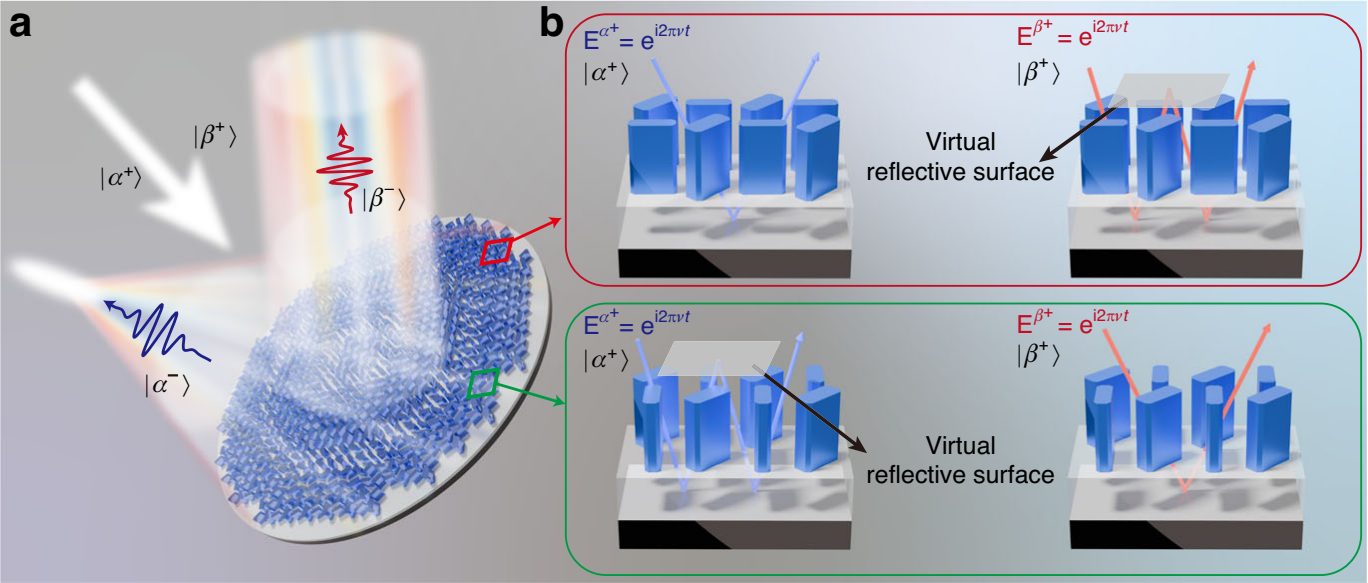
Principle of dispersion control based on folded-path metasurfaces. **a** Schematic of a folded-path metasurface that can achromatically deflect and focus broadband light for two orthogonal polarization (or spin) states independently. **b** Diagram of polarization-dependent light path folding through a virtual reflective surface generated by local interference. The left and right panels represent the cases of two orthogonal incident polarizations. The top and bottom panels represent different cases

As conceptually depicted in Fig. 1a, the proposed folded-path metasurface enables polarization-decoupled dispersion control and wavefront shaping with subwavelength resolution. When the polarization state $|{\alpha }^{+}\rangle$ is incident, the reflected light achieves broadband achromatic focusing. Conversely, when the orthogonal polarization state $|{\beta }^{+}\rangle$ is incident, the reflected light achieves achromatic deflection. This capability is difficult to achieve with a traditional metasurface, as it demands not only independent phase control but also independent dispersion control of two orthogonal polarization (or spin) states.

In general, optical elements will introduce frequency-dependent propagation phases that can be written as^35,53^:

1 $$\varphi (\nu )=\frac{2\pi \nu }{c}{n}_{{\rm{e}}}(\nu )h$$

where *n*_e_, *ν*, and *h* are the effective refractive index, frequency, and path length, respectively. Dispersion control hinges on designing nanostructures with the necessary group delay given by:

2 $$\frac{\partial \varphi }{\partial \nu }=\frac{2\pi }{c}{n}_{{\rm{e}}}(\nu )h+\frac{2\pi \nu }{c}\frac{\partial {n}_{{\rm{e}}}(\nu )}{\partial \nu }h$$

The emphasis in dispersion control lies in adjusting the effective refractive index or path length. Usually, metasurfaces regulate the effective refractive index by altering the structural size; however, this method struggles to simultaneously achieve independent dispersion control and wavefront shaping for arbitrary orthogonal polarizations, as their effective refractive index and propagation phases are inherently coupled.

To solve this issue, we propose a concept of folded-path metasurfaces enabled by local interference to realize polarization-decoupled control of equivalent path length, while maintaining an unchanged structure height. As illustrated in Fig. 1b, the metasurface is composed of nanopillars on a dielectric gap and a metal film. To facilitate understanding, we consider the nanopillars and gap as a unified entity. When the local interference occurs at a subwavelength scale and exhibits destructive interference^60^, it can effectively create a virtual reflective surface near the exit surface of the nanopillars.

Figure 1b displays two different scenarios, corresponding to the red box, and green box depicted in Fig. 1a. Here, to more clearly illustrate the multiple reflections of light, we represent the normally incident light on the metasurface as tilted rays. For example, consider the structure within the red box. When the polarization state $|{\alpha }^{+}\rangle$ is incident, light reflects off the substrate once before returning to the air (left panel of the red box in Fig. 1b). However, for the orthogonal polarization state $|{\beta }^{+}\rangle$, destructive interference leads to the formation of a virtual reflection surface, causing the light path to fold repeatedly between the substrate and this virtual reflection surface (right panel of red box in Fig. 1b). The light therefore requires two reflections from the substrate to return to the air, which increases the effective path length and consequently increasing the group delay. In contrast, for the structure within the green box, when the polarization state $|{\alpha }^{+}\rangle$ is incident, the path of the incident light is folded (left panel of the green box in Fig. 1b), as opposed to the situation in the red box. The conversion between these two cases is achieved by polarization-decoupled local interferences among adjacent nanopillars.

**Fig. 2:**
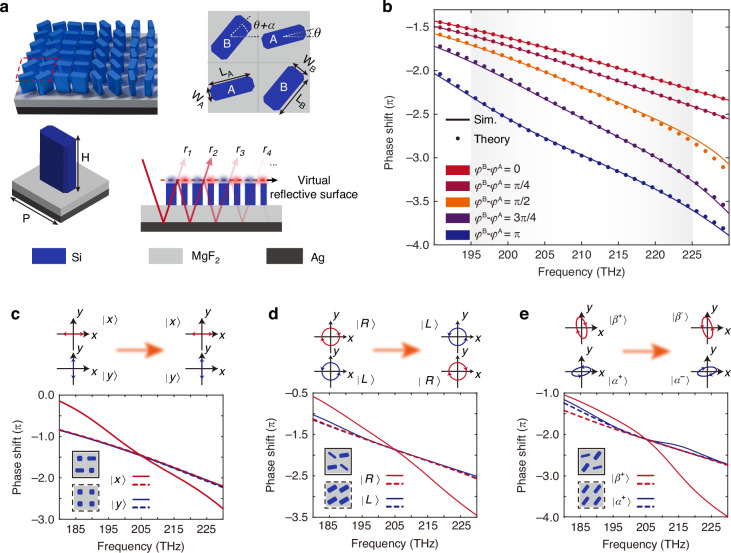
Demonstration of independent dispersion control on an arbitrary pair of orthogonal states of polarization. **a** Diagram of supercell array (red dashed box) consisting of four anisotropic nanopillars. **b** Comparison of the phase spectra obtained from the multiple-reflection model and full-wave simulation, verifying the tunability of dispersion based on the phase differences between subcells A and B. **c–e** Phase spectra for different orthogonal polarization combinations. Blue and red curves indicate the results for two orthogonal polarization states. Solid and dashed curves correspond to the results obtained by two different supercells shown in the inset

To implement such polarization-decoupled local interferences, we employ supercell design in the near-infrared range. As shown in Fig. 2a, it consists of a pair of staggered twin nanopillars (A and B) and forms a local interference system. The subcells consist of silicon nanopillars, magnesium fluoride (MgF_2_) gap, and silver (Ag) film. The nanopillars, each with a height of *H* = 450 nm, are arranged in a square array with a period of *P* = 500 nm. The incident light passes through the nanopillars and dielectric gap before being reflected by the metal film. Due to the negligible losses within the dielectric film, this reflective structure exhibits high reflectance (>95%). The simulation results for reflectance are available in section S1 of the Supplementary Information. Consequently, the output electric fields of the two subcells can be given as ${e}^{i{\varphi }_{\alpha /\beta }^{{\rm{A}}}}$ and ${e}^{i{\varphi }_{\alpha /\beta }^{{\rm{B}}}}$, respectively, where ${\varphi }_{\alpha /\beta }^{{\rm{A}}/{\rm{B}}}$ denotes the total phases carried on nanopillars A and B for two orthogonal polarizations $|{\alpha }^{+}\rangle$ and $|{\beta }^{+}\rangle$. The sizes of the supercell remain at the subwavelength scale, allowing the local interference of electromagnetic fields among subcells^18,61^. The final resultant intensity of the polarization-decoupled interference system is given by:

3 $${r}_{\alpha /\beta }=\frac{1}{2}\left[1-\,\cos \left({\varphi }_{\alpha /\beta }^{{\rm{B}}}-{\varphi }_{\alpha /\beta }^{{\rm{A}}}\right)\right]$$

The reflectivity depends on the phase difference between adjacent nanopillars. By adjusting this phase difference to create constructive or destructive interference, the virtual reflectivity can be controlled from 0 to 100%. Consequently, part of the reflected light passes through the virtual reflective surface and enters the air, while the remainder is reflected into the cavity formed by the supercell array and metal layer. As shown in the bottom right of Fig. 2a, the total reflection coefficient can be represented as the complex-amplitude superposition of multiple reflections (see section S1 of the Supplemental Information for details):

4 $${R}_{\alpha /\beta }={e}^{i{\varphi }_{\alpha /\beta }^{{\rm{A}}}}\frac{1+{e}^{i\left({\varphi }_{\alpha /\beta }^{{\rm{B}}}-{\varphi }_{\alpha /\beta }^{{\rm{A}}}\right)}-2{e}^{i\left({\varphi }_{\alpha /\beta }^{c}+{\varphi }_{\alpha /\beta }^{{\rm{B}}}\right)}}{2-{e}^{i\left({\varphi }_{\alpha /\beta }^{c}+{\varphi }_{\alpha /\beta }^{{\rm{A}}}\right)}-{e}^{i\left({\varphi }_{\alpha /\beta }^{c}+{\varphi }_{\alpha /\beta }^{{\rm{B}}}\right)}}$$

where ${\varphi }_{\alpha /\beta }^{c}$ denotes the additional phase experienced by the light reflected by the virtual reflective surface. Equation (4) assumes periodic boundary conditions. When the phase and dispersion gradients are small, the geometrical differences between adjacent supercells are negligible, making the approximate periodic boundary condition valid. Additionally, high-contrast dielectric nanopillars can act as weakly coupled, low-quality-factor resonators^62^, allowing each supercell to operate nearly independently. In such cases, the influence of neighboring supercells can be disregarded.

Initially, we validated the multiple-reflection model with five supercells formed from polarization-insensitive meta-atoms. These supercells are composed of unit structures with different phase differences. Figure 2b compares the phases obtained by the multiple-reflection model and the full-wave simulation for five different phase differences. The results demonstrate strong agreement between the multiple-reflection model and the full-wave simulation, confirming that adjusting the phase differences of the subcells enables dispersion control (see section S1 of the Supplemental Information for more details). According to the theory of composite phase control in asymmetric photonic spin–orbit interactions^18,20,63^, the total phase shift carried on each nanopillar is the sum of the propagation phase and geometric phase that are determined by the size and orientation of each nanopillar, respectively. By proper design, any combination of phase shifts can be achieved for two orthogonal polarizations. As a result, the wavefront and dispersion, which are determined by ${\varphi }_{\alpha /\beta }^{{\rm{A}}}$ and ${\varphi }_{\alpha /\beta }^{{\rm{B}}}-{\varphi }_{\alpha /\beta }^{{\rm{A}}}$ respectively, can be controlled independently. See section S2 of the Supplemental Information for details.

To further valid independent dispersion control for arbitrary orthogonal polarizations, we demonstrate two kinds of supercells for each pair of orthogonal linear, circular, and elliptical polarizations (*δ* = π/3, *χ* = π/6), as illustrated in Fig. 2c–e. In the linear polarization basis, arbitrary orthogonal polarizations can be expressed as^20^:

5 $$|{\alpha }^{+}\rangle =\left[\begin{array}{c}\cos \,\chi \\ \sin \,\chi {e}^{i\delta }\end{array}\right],\,|{\beta }^{+}\rangle =\left[\begin{array}{c}\sin \,\chi \\ -\,\cos \,\chi {e}^{i\delta }\end{array}\right]$$

where *δ* and *χ* indicate the ellipticity and azimuth angle of the polarization ellipse, respectively. The output polarization states $|{\alpha }^{-}\rangle$ and $|{\beta }^{-}\rangle$ are identical to the input states with reversed handedness.

For the first kind of supercells (solid box), when the polarization state $|{\alpha }^{+}\rangle$ is incident, the phase difference between nanopillars A and B is 0. However, for the polarization state $|{\beta }^{+}\rangle$, the phase difference is π, causing the light to be reflected by the virtual reflective surface. Therefore, the equivalent path length increases, resulting in a greater group delay, as illustrated by the red solid curves in Fig. 2c–e. Conversely, for the second type of supercells (dashed box), regardless of whether the incident polarization state is $|{\alpha }^{+}\rangle$ or $|{\beta }^{+}\rangle$, the phase difference between subcells remains 0, leading to nearly equal group delays, as depicted by the dashed curves. These results substantiate the independent dispersion control of two orthogonal polarizations. In addition, it should be noted that there are minor differences in the dashed lines shown in Fig. 2e. These discrepancies stem from the inherent dispersion of the propagation phase and the anisotropy in the nanopillars, resulting in a small deviation between the actual and ideal polarization conversion. More details of geometrical parameters are presented in section S2 of the Supplemental Information.

### Experimental demonstrations

**Fig. 3:**
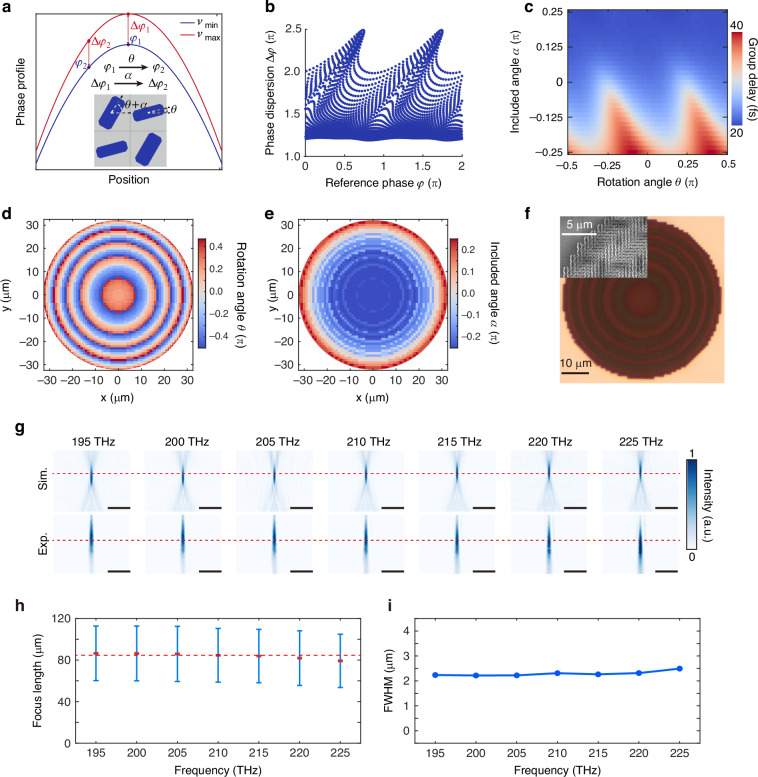
Broadband achromatic metalens simply by rotating nanopillars. **a** Phase distribution for the metalens at the maximum and minimum incident frequencies. The rotation angle *θ* and included angle *α* are used to control reference phase *φ* and phase dispersion Δ*φ*, respectively. **b** Simulated reference phase and phase dispersion for all combinations of *α* and *θ*. **c** Group delay as functions of *α* and *θ*. **d** Spatial distribution of the rotation angle *θ*. **e** Spatial distribution of the included angle *α*. **f** Optical and electron microscopy images of the sample. **g** Simulated and measured the intensity on the xoz plane at LCP incidence. **h** Measured focal length. The line segment represents the FWHM of the focal depth. **i** FWHM of the focal spots. Scale bar: 20 μm. FWHM full width at half maximum

Relying on the ability of flexible dispersion control, we first experimentally demonstrate an achromatic metalens within the frequency band of 195–225 THz. Different from earlier achromatic metalenses that require varying geometrical sizes, our designed metalens utilize a supercell with fixed dimensions. As illustrated in the inset of Fig. 3a, the sizes of the subcells are fixed at *L*_A_ = 430 nm, *W*_A_ = 150 nm, *L*_B_ = 450 nm, and *W*_B_ = 200 nm, with only the structural orientation angle changing. In specific, the propagation phase of the two types of subcells differs by π/2 at the frequency of 195 THz, and the rotation angles are *θ* and *θ* + *α*, respectively. As a result, the total phase difference between subcells A and B can vary from 0 to π as the included angle *α* changes from -π/4 to π/4. As shown in Fig. 3a, *φ* is considered the basic reference phase of the metalens, which is only related to the minimum frequency of *ν*_min_. This phase profile can be achieved using the dispersion-free geometric phase that is equal to ±2*θ*^57,64^, where the symbol depends on the spin state of the incident circularly polarized light. Δ*φ* is a function of working frequency and position, which requires dispersion control via changing the included angle *α*.

Figure 3b shows the simulated reference phase (the phase shift at the frequency of 195 THz) and phase dispersion (the phase difference between the frequencies of 195 THz and 225 THz) for a library of supercells with various combinations of *α* and *θ*. Simultaneous adjustments to both *θ* and *α* offer the capability for independent control over wavefront and dispersion. Simulated group delays as functions of *α* and *θ* are shown in Fig. 3c. This design strategy can be extended to enable achromatic metalenses with larger apertures by stronger dispersion control through increased structural heights^53,65^. To better match the phase distributions at different frequencies, a particle swarm optimization algorithm is employed to optimize the orientation distributions of nanopillars A and B. In Fig. 3d, e, we show the spatial distribution of the optimized parameters of the supercells forming the achromatic metalens. It can be observed that the rotation angle *θ* varies with the radius, consistent with the change in geometric phase. Figure 3f shows optical and electron microscopy images of the sample. Under the illumination of left-handed circularly polarized (LCP) light, the simulated and measured light intensity distributions on the xoz plane are illustrated in Fig. 3g, demonstrating an almost perfect achromatic focusing ability. Figure 3h displays the experimentally measured focal length, with the line segment representing the full width at half maximum (FWHM) of the focal depth, and the red dots marking the center of the focal depth. It is evident that good achromatic performance is maintained within the frequency range of 195 THz to 225 THz. The FWHM of the focal spot was measured in the xoy plane where the maximum light intensity occurs, with the results presented in Fig. 3i. The measured values are slightly larger than the theoretical values. This discrepancy arises because a focused beam was used as the incident light during the characterization of the metalens focusing performance, slightly affecting the focal spot. More details of the design and results are provided in Section S3 of the Supplemental Information.

**Fig. 4:**
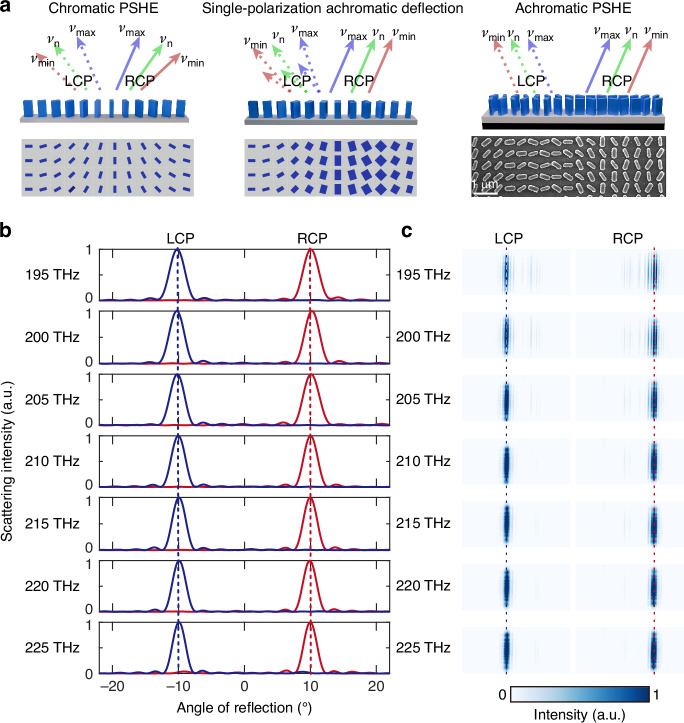
Broadband achromatic PSHE simply by rotating nanopillars. **a** Comparison among classical chromatic PSHE (left), single-polarization achromatic deflection (middle), and broadband achromatic PSHE (right). **b** Simulated far-field intensity profiles at various incident frequencies. The reflection angles keep at around −10 and +10 degrees at LCP and RCP incidence, respectively. **c** Captured intensity images of scattering light for different incident frequencies

Building on the independent dispersion control of two orthogonal polarizations, we have achieved a broadband achromatic PSHE, which is unprecedented. This is specifically depicted in Fig. 4a. It demonstrates PSHE with chromatic aberration, single-polarization deflection for chromatic aberration correction, and broadband achromatic PSHE. In the first case, the reflection angle of LCP light varies with different incident frequencies, and the same applies to right-handed circularly polarized (RCP) light. In the second case, the reflection angle of LCP light remains consistent across different incident frequencies, whereas RCP light exhibits random scattering owing to unmatched dispersion compensation. In the third case, the reflection angles for both LCP and RCP light remain almost unchanged when the incident frequency varies from 195 THz to 225 THz. Unlike previous methods that achieved achromatism for a single spin state, the innovative design extends this capability to encompass two opposite spin states.

Figure 4b displays the simulated far-field intensity profiles for LCP and RCP incidence. The far-field intensity distribution reveals that the primary energy is concentrated in the main lobe, with virtually no side lobes, which confirms that the electromagnetic crosstalk between adjacent supercells is negligible. The reflection angles for both LCP and RCP show minimal variation across a broad frequency range, illustrating broadband achromatic performance. This is further validated experimentally by capturing scattered light through a slight focus, as shown in Fig. 4c. The beam spot remains centered on a detector while the incident frequencies fluctuate, aligning well with the numerical simulation. The building blocks and design methodology are the same as those used in the above achromatic metalens (see section S4 of the Supplemental Information for more details).

## Discussion

The ability to independently control dispersion and phase for two orthogonal polarizations can further be applied to create spatiotemporal vector optical fields based on a single metasurface. Current spatiotemporal optical field manipulation typically requires two separate Fourier transform setups to independently control the phase, amplitude, and dispersion of two orthogonal polarization components, resulting in a complex and bulky system^46–48,59^. Initially, one can generate time-varying polarization states by leveraging the different group delays of LCP and RCP (Fig. S11). It is noteworthy that, unlike previously achieved time-varying polarization states, this method avoids the complex optical systems of Fourier synthesis.

**Fig. 5:**
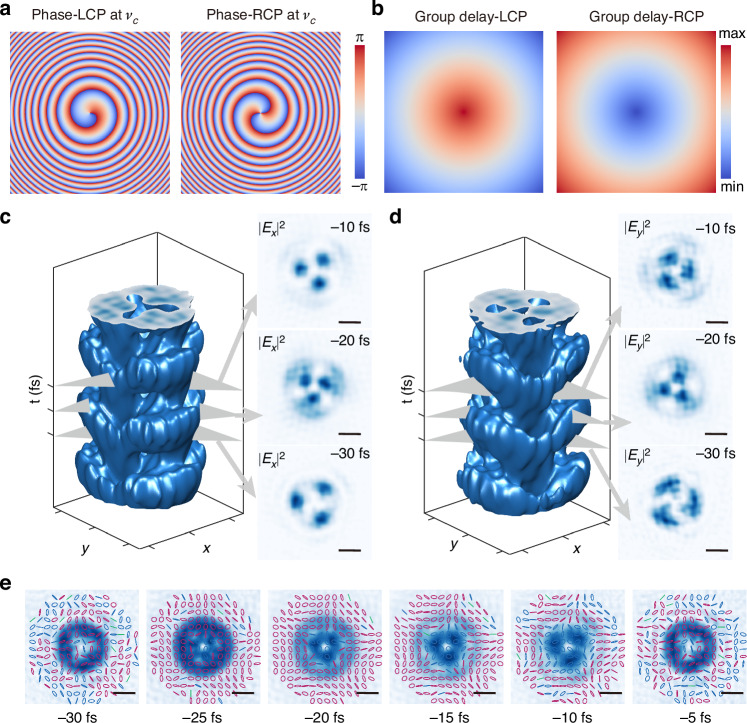
Spatiotemporal vector optical field generated by a single metasurface carrying azimuthal-varied vortex phase and radial-varied dispersion. **a** Phase distributions of LCP and RCP at the center frequency. **b** Group delay distributions of LCP and RCP at the center frequency. **c**, **d** Spatiotemporal wave packet of *E*_*x*_ and *E*_*y*_ components. The plot shows the iso-intensity profile at 10% of the peak intensity. Inset: instantaneous intensity distribution at three time delays *t* = −30 fs, −20 fs, and −10 fs. **e** Evolution of the optical vector fields over time. Right-handed, left-handed, and near-linear polarizations are represented by red, blue, and green colors. Scale bar: 2 μm

To verify the ability of spatiotemporal vector optical field manipulation, we design a metasurface with polarization-dependent vortex phase and radial-varied dispersion, as shown in Fig. 5a, b. The temporal displacement between the LCP and RCP components causes the polarization azimuth angle of the output light to vary over time when linearly polarized light is incident. The synthesized field exhibits time-periodic polarization and intensity distributions throughout the pulse duration. To the best of our knowledge, this is the first demonstration of spatiotemporal vector optical field manipulation using a single metasurface. Figure 5c, d shows the spatiotemporal wave packet components *E*_*x*_ and *E*_*y*_, respectively. The insets display the instantaneous intensity distribution at three representative time delays: *t* = −30 fs, −20 fs, and −10 fs. It can be observed that, over time, the spatial distribution of light intensity initially contracts and then diverges, driving a time-dependent change in the spatial distribution of the polarization state. The instantaneous polarization states of the synthesized spatiotemporal vector optical field at six different points in time are shown in Fig. 5e. See section S5 of the Supplemental Information for more details.

In summary, we propose the concept of folded-path metasurfaces, demonstrating that the dispersion and phase control of two opposite spin states can be independently and flexibly controlled by a single monolayer metasurface. This remarkable ability arises from breaking the reliance on dispersion-engineered metasurfaces on spin-coupled and phase-coupled effective refractive index control. Instead, local interference is utilized to fold the optical path, effectively modifying the equivalent path length to achieve dispersion control. Combined with composite phase modulation, it enables simultaneous spin-decoupled dispersion and phase control. To validate capabilities that traditional dispersion-engineered metasurfaces cannot achieve, we experimentally demonstrate broadband achromatic focusing and achromatic photonic spin Hall effect simply by rotating nanopillars. Additionally, a single metasurface is simulated to generate spatiotemporal vector optical fields without the need for complex and bulky Fourier transform setups.

This folded-path metasurface platform is set to unlock unprecedented opportunities for spin photonics, expanding the functionality of spin-decoupled metasurfaces beyond traditional spatial applications into the spatiotemporal domain. Notably, although this method was demonstrated in the near-infrared range, it can be adapted to other wavelength ranges by scaling the geometric dimensions. For instance, in the visible spectrum, MgF_2_ can be replaced with silicon dioxide (SiO_2_), and Si can be substituted by titanium dioxide (TiO_2_)^35^ or gallium nitride (GaN)^34^. We believe that a physics-data-driven optimization model, integrating adjoint shape optimization and deep learning, could further enhance the performance of the proposed folded-path metasurfaces for dispersion control^66,67^. A general outline of the technical approach and algorithmic steps is provided in section S8 of the Supplemental Information. This advancement paves the way for a wide range of innovations, from dynamic control of light-matter interactions to the development of next-generation spin-photonic devices, significantly broadening the scope and impact of metasurface technologies in both fundamental research and practical applications.

## Methods

### Simulations

The finite element method in CST Microwave Studio and Rigorous Coupled-Wave Analysis was employed to calculate the optical properties of the designed meta-atoms and metasurfaces. For period structures, the unit-cell boundary conditions were applied along the *x*- and *y*-direction. The electric boundary conditions and open boundary conditions were applied to the two ends in the z-direction, respectively. For the achromatic metalens, the electromagnetic field was first computed 1 μm above the structure in CST Microwave Studio. The focusing performance was then evaluated using vector angular spectrum theory^57^. For the simulation, the permittivity of MgF_2_ is assumed to be 1.88, and the permittivity of Ag is sourced from Johnson’s handbook^68^. Further simulation details on the spatiotemporal field are included in section S5 of the Supplemental Information.

### Fabrications

The fabrication process of the sample is shown in Figure S12. First, the chromium layer, silver layer, and magnesium fluoride layer are sputtered on a cleaned silicon wafer by magnetron sputtering, where the thickness of the silver layer is greater than 150 nm, and the thickness of the magnesium fluoride is 250 nm. The chromium layer serves to improve adhesion. Subsequently, 450 nm of silicon is deposited on the surface of the magnesium fluoride layer by plasma-enhanced chemical vapor deposition. In the next step, a photoresist (maN2401) with a thickness of about 100 nm was spin-coated on the surface of the silicon layer. Afterward, the pattern is transferred to the photoresist layer by electron beam lithography (125 kV), the pattern is formed by inductively coupled plasma etching, and the processed sample is obtained by removing the residual photoresist.

### Characterizations

As shown in Fig. S13, the sample was illuminated with a focused laser beam. Prior to illuminating the sample, the laser beam was passed through a linear polarizer, a quarter-wave plate, and a beam splitter. The linear polarizer and quarter-wave plate were used to control the polarization state of the incident light. The reflected light was then collected by a microscope objective lens and re-imaged onto an image sensor using a tube lens. To manipulate the polarization of the reflected light, a quarter-wave plate and a linear polarizer were positioned between the objective lens and tube lens. For characterizing the metalens focusing performance, the objective lens had a numerical aperture (NA) of 0.7, the tube lens had a focal length of 200 mm, and the system magnification was 100×. The laser used for illumination was a supercontinuum spectrum laser.

## Supplementary information

Supplementary Information for Dispersion-engineered spin photonics based on folded-path metasurfaces

## Data Availability

The data that support the findings of this study are available from the corresponding author upon request.
