# Supplementary material for: Dispersion-engineered spin photonics based on folded-path metasurfaces
Source: Light Sci Appl. 2025 May 16;14:198. doi: 10.1038/s41377-025-01850-w (PMC12084559; doi:10.1038/s41377-025-01850-w)
Supplement: Supplementary file 1 — Supplementary Information for Dispersion-engineered spin photonics based on folded-path metasurfaces [file 41377_2025_1850_MOESM1_ESM.docx]

Supplementary Information for

**Dispersion-engineered spin photonics based on folded-path metasurfaces**

Fei Zhang^1,2,3,4^,^†^, Hanlin Bao^1,2,3,†^, Mingbo Pu^1,2,3,4,*^, Yinghui Guo^1,2,3,4^, Tongtong Kang^1,4^, Xiong Li^1,2,3^, Qiong He^1,2^, Mingfeng Xu^1,2,3,4^, Xiaoliang Ma^1,2,3^, Xiangang Luo^1,2,3^,^*^

^1^ National Key Laboratory of Optical Field Manipulation Science and Technology, Institute of Optics and Electronics, Chinese Academy of Sciences, Chengdu 610209, China

^2^ State Key Laboratory of Optical Technologies on Nano-Fabrication and Micro-Engineering, Institute of Optics and Electronics, Chinese Academy of Sciences, Chengdu 610209, China

^3^ College of Materials Sciences and Opto-Electronic Technology, University of Chinese Academy of Sciences, Beijing 100049, China

^4^ Research Center on Vector Optical Fields, Institute of Optics and Electronics, Chinese Academy of Sciences, Chengdu 610209 China

^†^ These authors contributed equally to this work.

^*^ Corresponding author. Email: pmb@ioe.ac.cn (M.B.P); lxg@ioe.ac.cn (X.G.L)

**This PDF file includes:**

Supplementary Text

Figs. S1 to S14

Tables S1

## S1. Multiple reflections model

The electric field responses of subcells A and B are respectively:

According to the coherent interference theory of light, the electric field response of the supercell consisting of subcells A and B is:

From Eq. S2, it can be seen that changing the value can change the single reflectivity of the supercell. A portion of the light is reflected into the cavity and is reflected again to the virtual reflective surface, and accordingly, the reflected complex amplitudes of the subcells are:

where *φ*_c_ denotes the phase during reflection, and we can express the complex amplitude of each reflection into the air using the recursive formula:

where *m* denotes the number of times it is reflected by the metal layer, and from the recursive equation (Eq. S4), the complex amplitude of rotation expressed in steps for each reflection into the air is obtained as:

The final total complex amplitude is the superposition of the complex amplitudes of the sub-levels:

As shown in Fig. S1A, when *φ^B^−φ^A^* = 0, the light is directly reflected into the air. Conversely, when *φ^B^−φ^A^* = π, the light is reflected by the virtual reflection surface into the nanopillars. The reflectivity of the virtual reflection surface can be controlled by adjusting the phase difference. Through successive reflections of light, the equivalent propagation distance increases, thereby enhancing the group delay. Figure S1B shows the comparison of the phases obtained by the multiple reflection model with those obtained by the full-wave simulation for five different phase differences, and it can be seen that the results of the multiple reflection model are in good agreement with those of the full-wave simulation. The parameters of the supercells corresponding to five different phase differences are shown in Fig. S1. Fig. S2 presents the reflectance of both subcells and supercells.


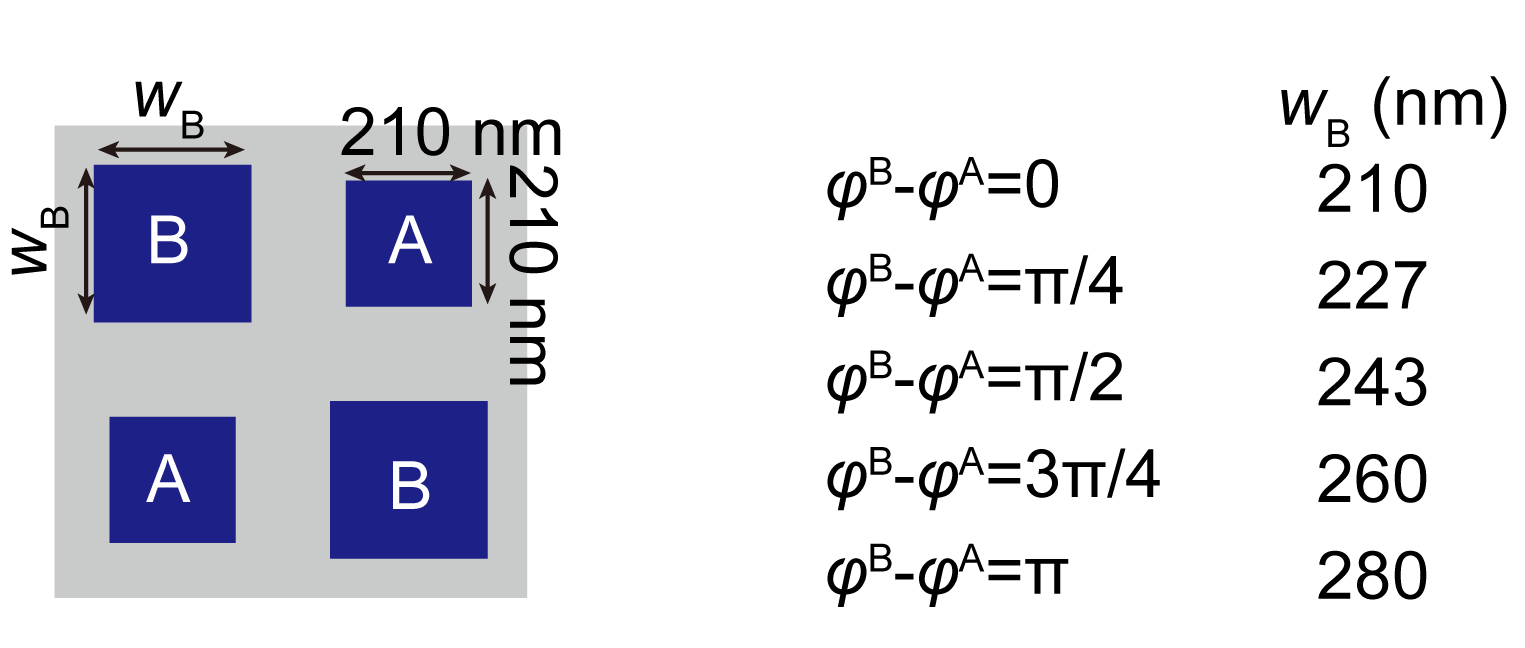


Fig. S1: Parameters of the supercells correspond to five different phase differences.


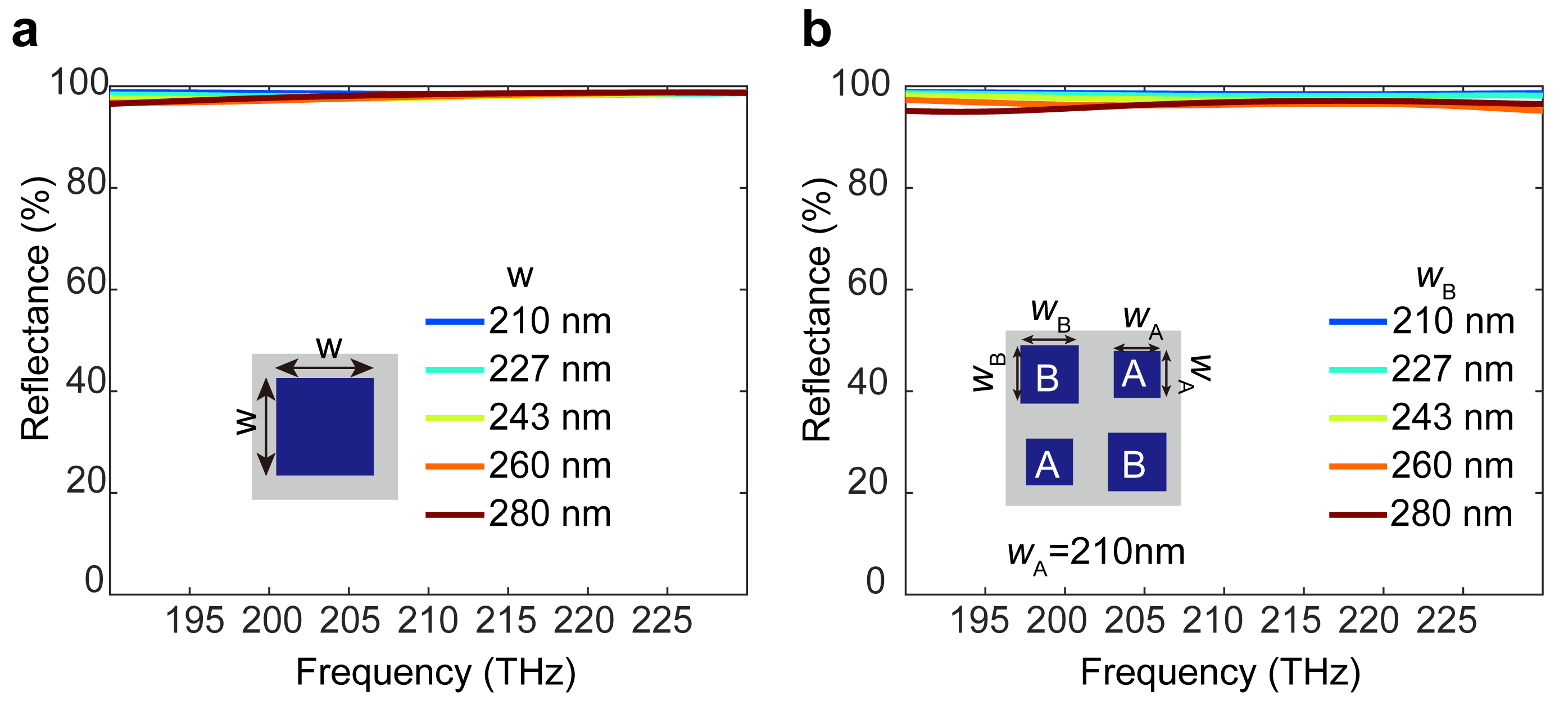


Fig.S2: Reflectance of different subcells (a) and supercells (b).

## S2. Composite phase control of the supercell

From Eq. S6, it is evident that when the phase and phase difference of subcells can be independently controlled, independent manipulation of wavefront and dispersion can be achieved. Here, we choose to use nanopillars with rotation angles to achieve independent control of the phase and phase differences of the subcells. The total phase carried on each nanopillar is the sum of the propagation phase and geometric phase that are determined by the size and orientation of each nanopillar, respectively. The composite phase control of the supercell can be described using the Jones matrix. Assume that the input polarization is represented as follows:

$$|\alpha^{+}\rangle=\left[\begin{matrix} \cos\chi\\ \sin\chi e^{i\delta} \end{matrix}\right];|\beta^{+}\rangle=\left[\begin{matrix} \sin\chi\\ -\cos\chi e^{i\delta} \end{matrix}\right]$$

where χ and δ represent the azimuth and ellipticity of the polarization ellipse, respectively. Then the output polarization can be written as:

$$|\alpha^{-}\rangle=\left[\begin{matrix} \cos\chi\\ \sin\chi e^{-i\delta} \end{matrix}\right];|\beta^{-}\rangle=\left[\begin{matrix} \sin\chi\\ -\cos\chi e^{-i\delta} \end{matrix}\right]$$

Then, the relationship between input polarization, output polarization, and the Jones matrix is satisfied as follows：

The expression for the Jones matrix can be written as:

where, representing the phase imparted to the polarization state $\{|\alpha^{-}\rangle,|\beta^{-}\}$by the A/B units within the supercell. *J^A^* (*x*, *y*) and *J^B^* (*x*, *y*) are symmetric unitary matrices. The rotation angle and propagation phase of the subcells can be obtained through matrix diagonalization:

where *φ_x_* and *φ_y_* are the propagation phases for linear polarized light along the fast and slow axes, respectively. The propagation phase and geometric phase are determined by the size and orientation of each nanopillar. Therefore, by varying the sizes and rotation angles of the two subcells (A and B), independent control and can be achieved. This facilitates the independent manipulation of both dispersion and wavefront for an arbitrary set of orthogonal polarizations. Table. S1 shows the detailed structural parameters of the proposed supercells. The original, unmodified phase envelopes are shown in the Fig. S3.

Table.S1: The supercell designs for the demonstration of the tunability of independent dispersion control. LP: linear polarization. CP: circular polarization. EP: elliptical polarization. Subscript 1: the first kind of supercells (solid box). Subscript 2: the second kind of supercell (dashed box)

|  | *L*_A_ (nm) | *W*_A_ (nm) | *L*_B_ (nm) | *W*_B_ (nm) | *θ* (π) | *α*(π) |
| --- | --- | --- | --- | --- | --- | --- |
| LP-1 | 365 | 170 | 210 | 215 | 0 | 0 |
| LP-2 | 210 | 215 | 210 | 215 | 0 | 0 |
| CP-1 | 420 | 160 | 420 | 100 | 0.028 | 0.810 |
| CP-2 | 420 | 190 | 420 | 190 | 0.141 | 0 |
| EP-1 | 400 | 165 | 400 | 105 | 0.295 | -0.222 |
| EP-2 | 150 | 400 | 150 | 400 | 1.795 | 0 |


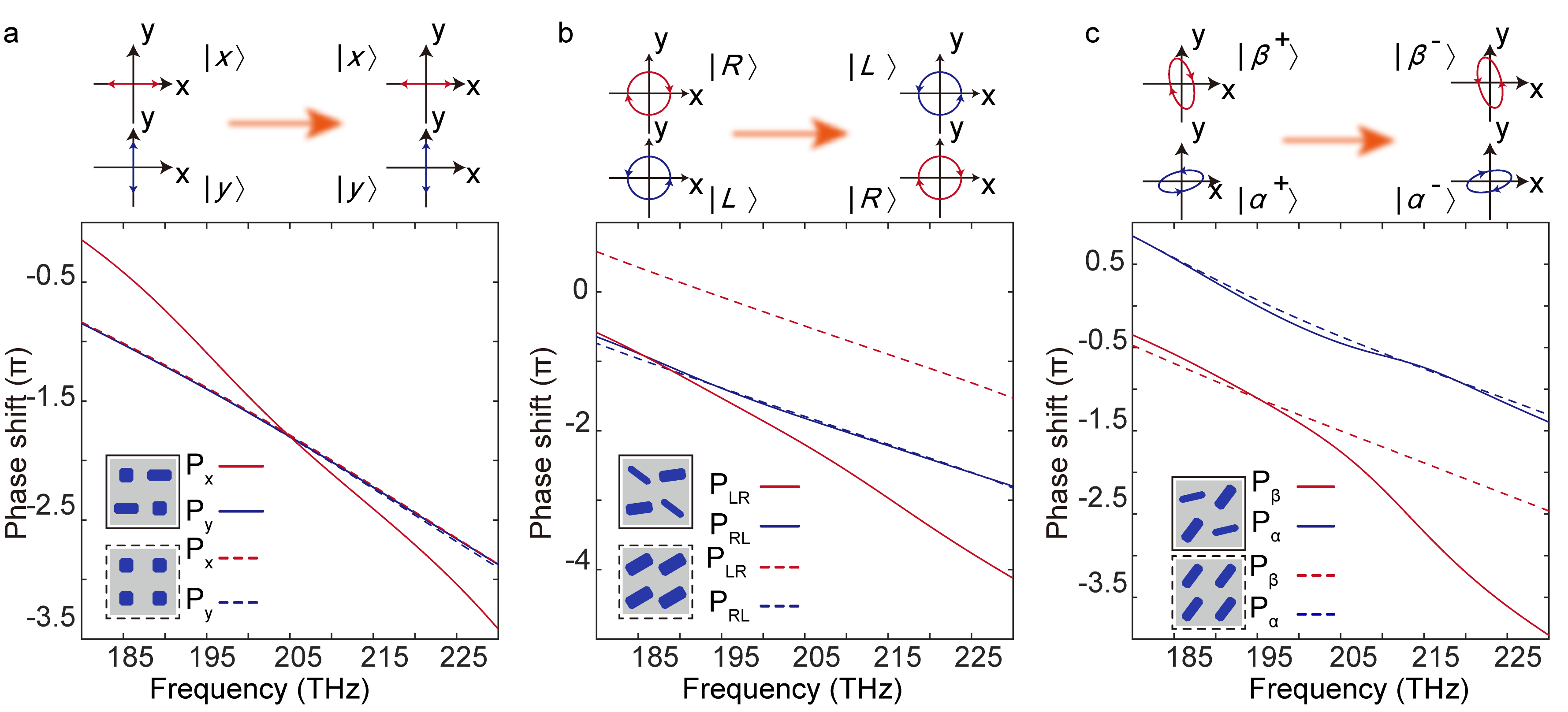


Fig. S3: Phase spectra for orthogonal polarization states show the tunability of the group delay by changing the sizes and orientations of supercells, including (a) linear polarization, (b) circular polarization, and (c) elliptical polarizations (δ = π/3, χ =π/6). Blue and red curves indicate the results for two orthogonal polarization states. Solid and dashed curves correspond to the results obtained by two different supercells schematically shown in the inset.

## S3. Broadband achromatic metalens

The ideal phase required at each frequency for a broadband achromatic metalens is given by Eq. S12:

where *ν* represents the frequency, c represents the speed of light, (x, y) represents Cartesian coordinates, and *f* stands for the focal length. The first part of *φ*_ν_ can be achieved using the geometric phase controlled by the rotation angle. The second part Δ*φ*_ν_ is a function of working frequency and presents a linear relation with *ν*, which is considered as the phase difference between various incident frequencies. Δ*φ*_ν_ can be written as:


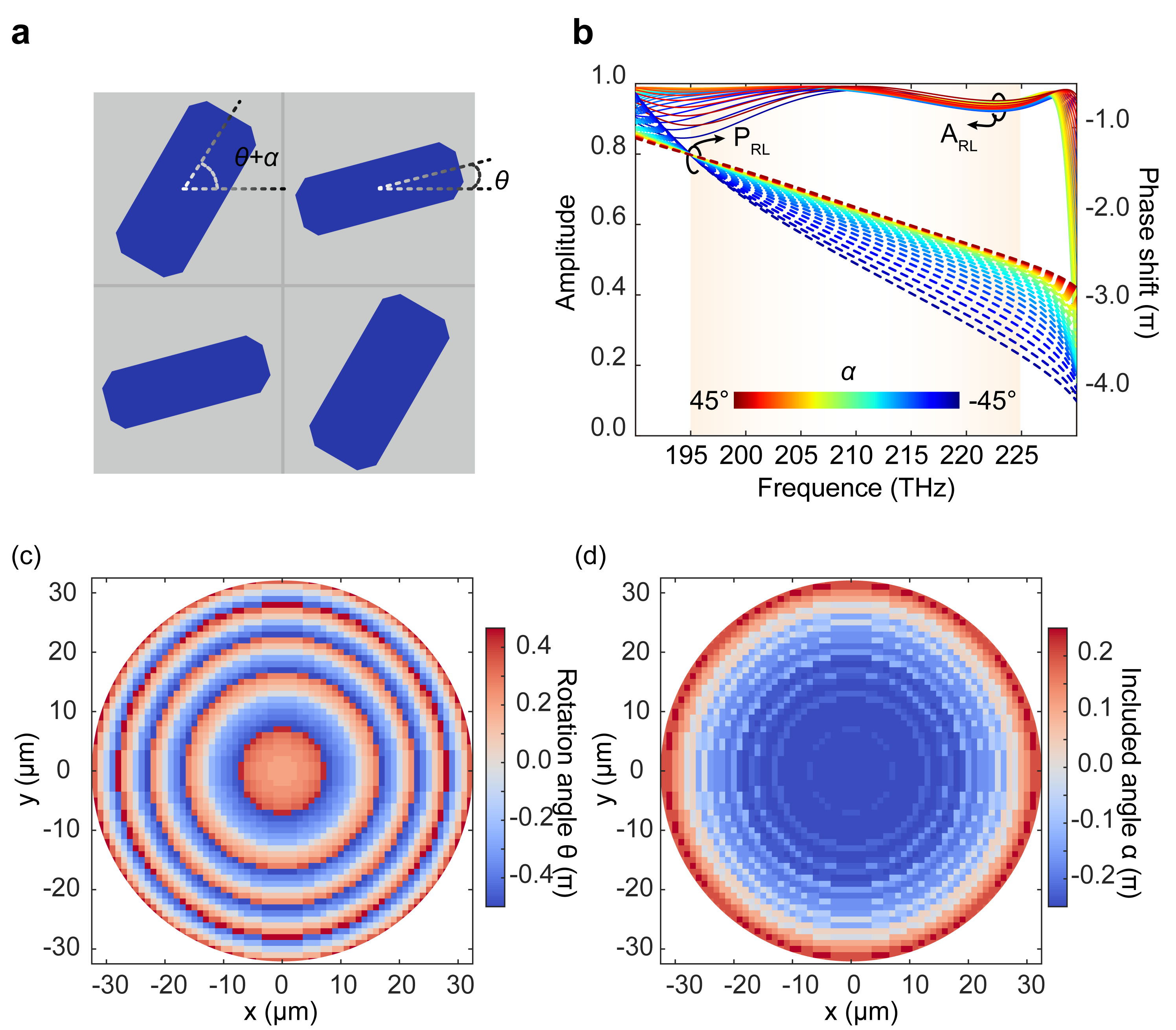


Fig. S4: Design of the achromatic metalens. **a** Schematic of the supercell including two adjustable parameters of *θ* and *α*.**b** Amplitude and phase profiles of the supercell vary with the angle *α*.

*C_ν_* denotes an optimizable constant that does not impact the focusing effect. Through optimizing *C*_ν_, Δ*φ*_ν_ can be adjusted to approximate the group delay attainable by the supercell. We used the particle swarm optimization method to optimize C_ν_ and find the most suitable unit structure for phase and group delay. In the design of the broadband achromatic metalens, we used the supercells shown in Fig. S4a, with the only adjustable parameters being *θ* and *α*. Figure S4b shows the simulated amplitude and phase profiles of the supercell with different angles *α.* Note that, the phase envelopes are shifted to better visualize the differences, and the phase shift can be achieved by adjusting *θ*. In Fig. S5, the optimized focusing phase is presented. Additionally, in Fig. S6, we present a comparison between the ideal focusing phase and the phase of the obtained structure, showing a good agreement. Figure S7 shows the simulation results, including the focal length, the full width at half maximum (FWHM) of the focal point, and the focusing efficiency, and the dashed line in Fig. 7a represents the theoretical results. It can be seen that the theoretical results are in good agreement with the simulation results. The simulated focusing efficiency is defined as the ratio of the power focused into the desired region to the input power, the desired region’ radius is three times the full width at half maximum of the focal spot.


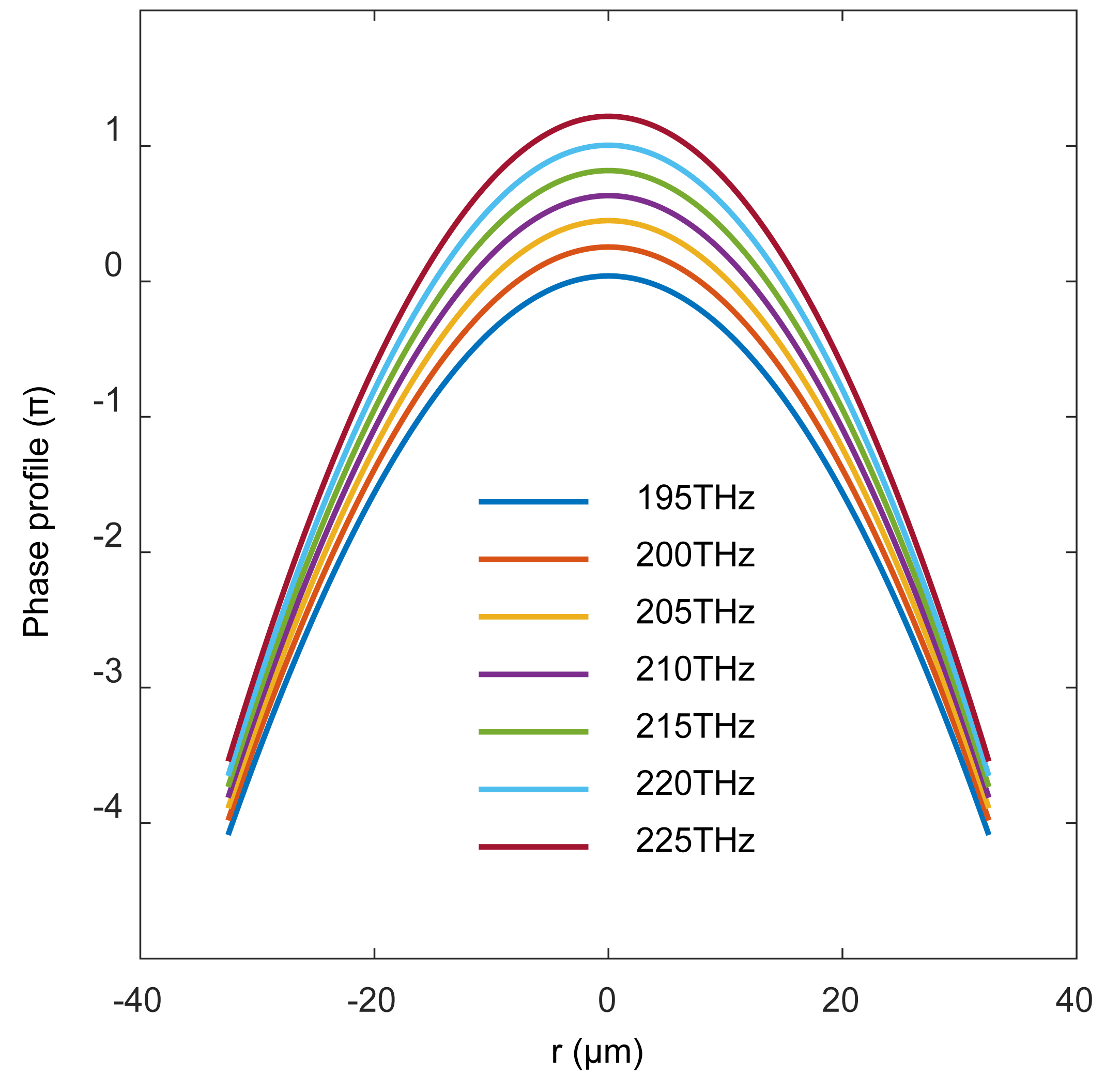


Fig.S5: Optimized focusing phases.


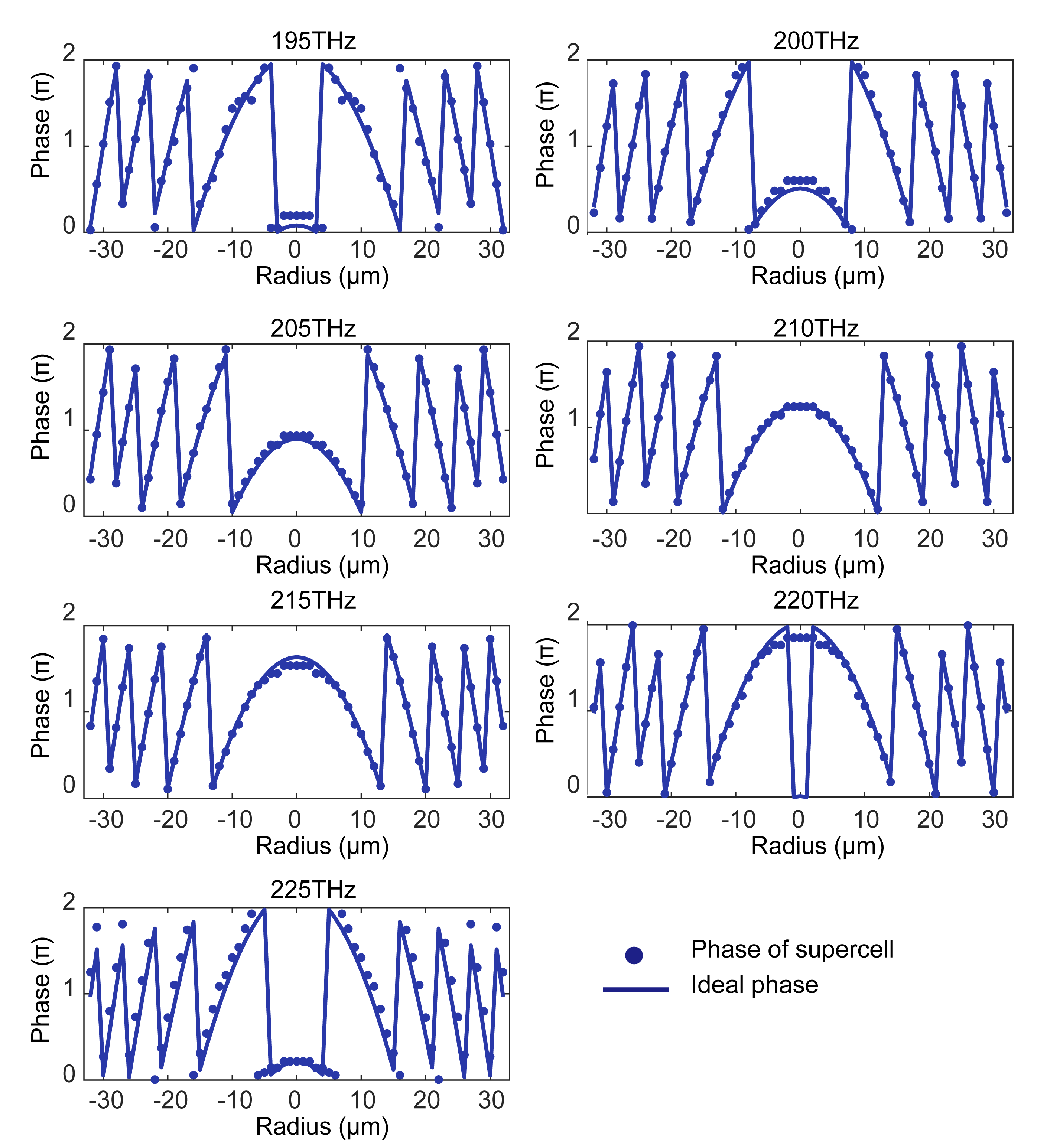


Fig. S6: Comparison between ideal and actual phase profiles of the achromatic metalens at different frequencies, where the solid lines represent the ideal wavefront phase profiles and the scatters denote the phases of the matched supercells.


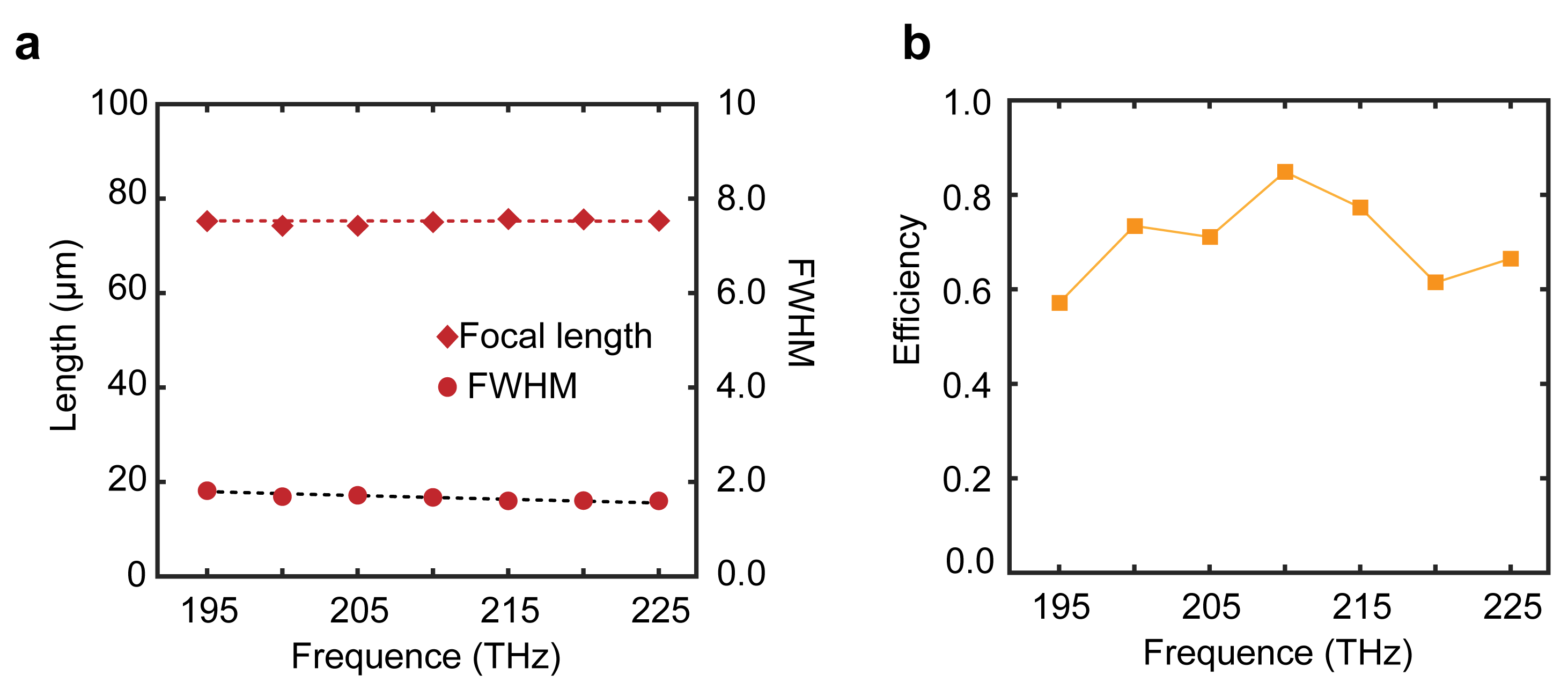


Fig. S7: Simulated focal length, full width at half maximum of focus point, and focusing efficiency.

## S4. Achromatic photonic spin Hall effect

The ideal phase required at each frequency for achromatic photonic spin Hall effect is given by Eq. S14:

where *ν* represents the frequency, *c* represents the speed of light, *x* represents the coordinate along the x-axis, and *γ* stands for the f diffraction angle. *σ* is 1 or -1, representing left-handed circular polarization (LCP) incidence or right-handed circular polarization (RCP) incidence, respectively. The first part of *φ*_ν_ can be achieved using the geometric phase controlled by the rotation angle. The second part Δ*φ*_ν_ is a function of working frequency and presents a linear relation with *ν*, which is considered as the phase difference between various incident frequencies. Δ*φ*_ν_ can be written as:

where *C_ν_* denotes an optimizable constant that does not impact the effect. Through optimizing *C_ν_*, Δ*φ*_ν_ can be adjusted to approximate the group delay attainable by the supercell.

Similar to the design of achromatic metalens, in achieving the achromatic photonic spin Hall effect, we used the particle swarm optimization method to find the most suitable supercells for phase and group delay. In Fig. S8, we show the spatial distribution of the parameters of the obtained supercells. Additionally, in Fig. S9, we present a comparison between the ideal phase and the phase of the obtained supercells, demonstrating that the phase of the optimized supercell matches well with the ideal phase.


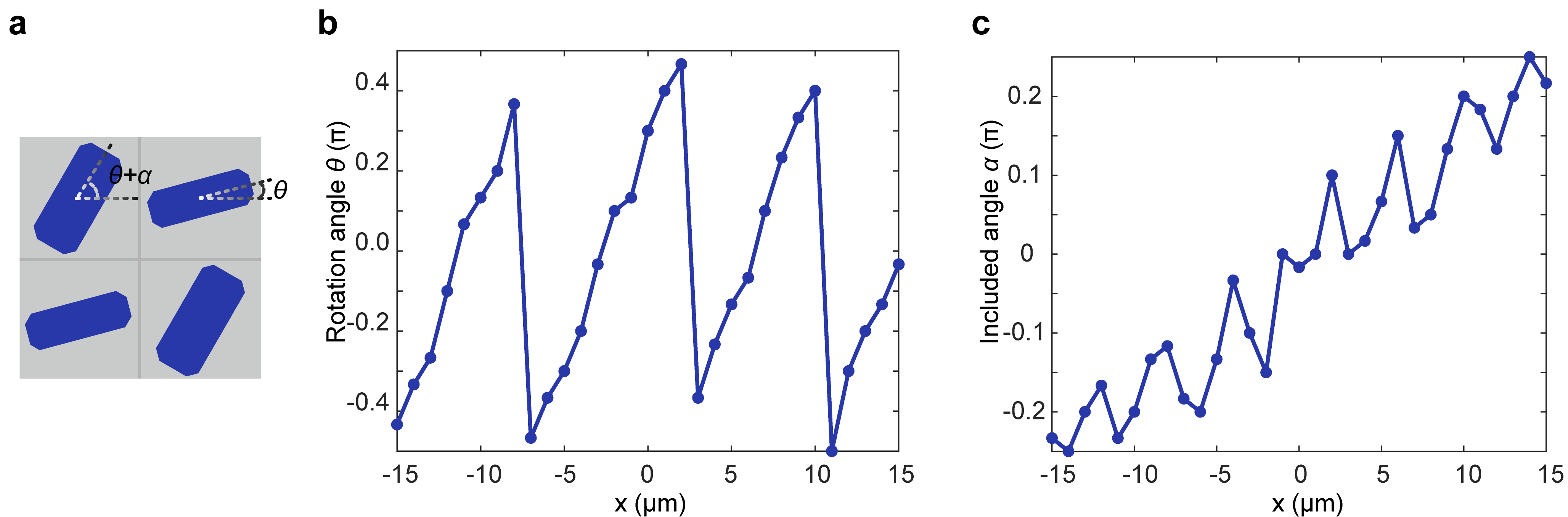


Fig. S8: Spatial distribution of the parameters of the supercell achieving the achromatic Hall effect. **a** Schematic of the supercell including two adjustable parameters of *θ* and α. **b** Spatial distribution of rotation angle *θ*. **c** Spatial distribution of included angle *α*.


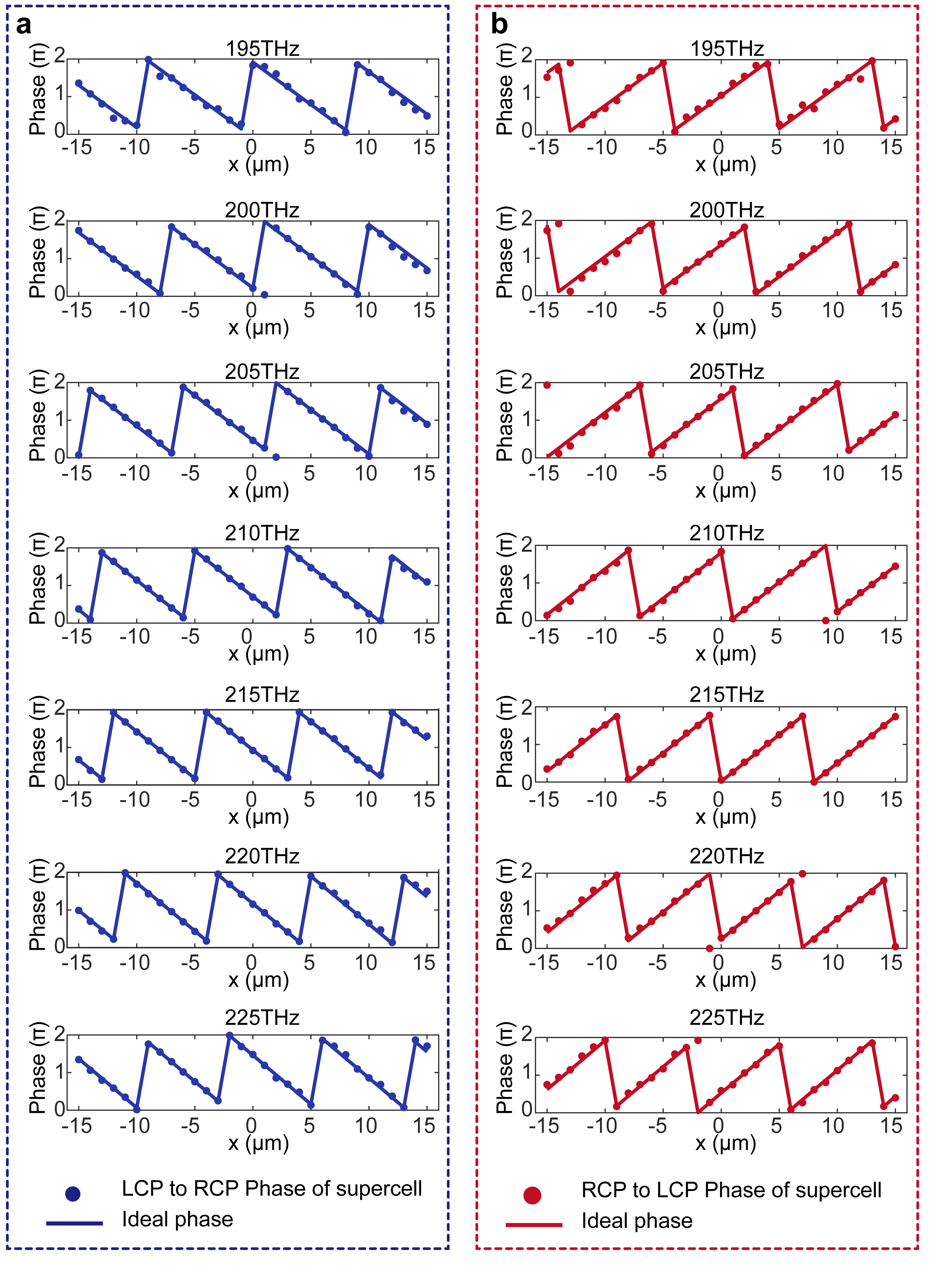


Fig. S9: Comparison between ideal and actual phase profiles for achieving the achromatic photonic spin Hall effect at different frequencies, where the solid lines represent the ideal wavefront phase profiles and the scatters denote the phases of the matched supercells. **a** LCP to RCP. **b** RCP to LCP

## S5. Spatiotemporal optical field manipulation

The manipulation of spatiotemporal optical fields has garnered significant research attention due to the significant potential for applications in ultrafast optics, nonlinear optics, and light-matter interactions. Manipulation of dispersion, polarization, and phase is the key to achieving spatiotemporal vector optical field manipulation. Since wavefront control and dispersion modulation are difficult to achieve with a single conventional optical element, most spatiotemporal optical field manipulation systems are based on Fourier synthesis methods, in which gratings and lens are used to perform the Fourier transform and inverse Fourier transform, a phase mask placed at the Fourier plane modulates the spatially separated spectral components of the input pulse. Here, using the ability of independent dispersion and phase control on two orthogonal polarizations, spatiotemporal optical field manipulation is demonstrated by a single metasurface. As shown in Fig.S10, the initial input pulse is:

$$E=\exp(-\frac{t^{2}}{2T_{0}^{2}}-i2\pi\nu_{c}t)$$

where *T*_0_ = 40 fs is the pulse width, and *ω_c_*=2π*ν_c_*, *ν_c_* denotes the center frequency. The phase applied to each frequency component by the metasurface for LCP and RCP components can be expressed as:

where$\beta_{1}^{\text{L/R}}$ and $\beta_{2}^{\text{L/R}}$ denote the group delay and group delay dispersion that determine the temporal displacement and pulse broadening, respectively. After undergoing modulation by the metasurface, the pulse is converted to:

$$E^{\text{L/R}}=\frac{T_{0}}{(T_{0}^{2}-i\beta_{2}^{\text{L/R}}z)^{1/2}}\exp\left(-i2\pi\nu_{c}t-\frac{(t-\beta_{1}^{\text{L/R}})^{2}}{2(T_{0}^{2}-i\beta_{2}^{\text{L/R}}z)}\right)$$

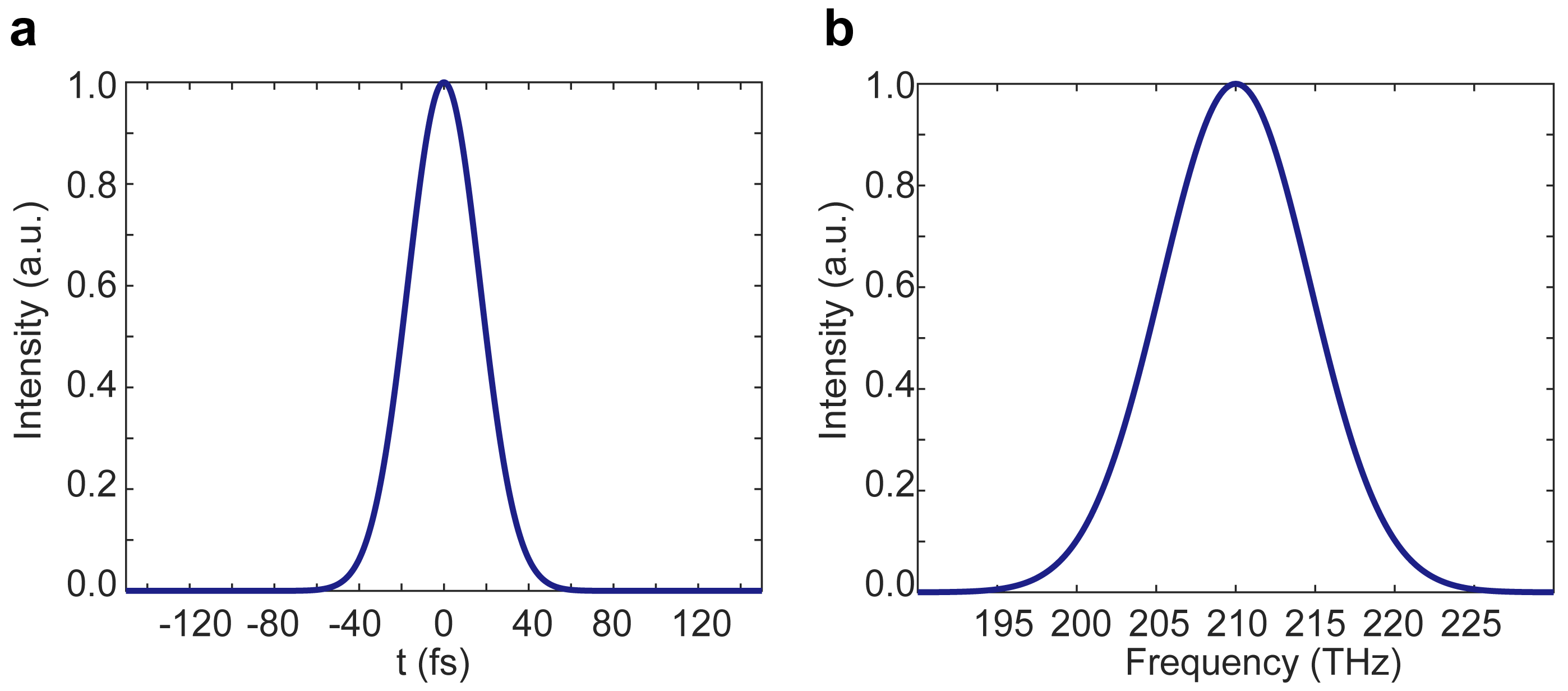


Fig. S10: Simulated input pulse. **a** Input pulse with duration ≈ 40 fs. **b** Frequency spectrum of the input pulse.

From Eq. , it can be seen that the dispersion differences between LCP and RCP lead to pulse separation in the time domain. As shown in Fig. S11, the incident linearly polarized pulse light is modulated by the metasurface, resulting in the separation into RCP and LCP pulses. Analyzing in the frequency domain, when broadband x-linearly polarized light impinges on the metasurface, the LCP and RCP components experience different group delays in the outgoing light, and the phase difference between LCP and RCP in the outgoing light varies with frequency, showing frequency dependence of the polarization state (middle panel of Fig. S11). In the time domain, the separation of LCP and RCP components over time results in the polarization state changing with time. Furthermore, by adjusting the rotation angle α, the frequency-dependent phase difference between LCP and RCP can be modified, thereby altering the trajectory of the polarization state on the Poincaré sphere with frequency. When α=45°, the polarization state on the Poincaré sphere in the frequency domain changes from x-polarized to nearly y-polarized in a counterclockwise direction, while in the time domain, the polarization state transitions from left-handed to right-handed. When α=0°, since the group delays of LCP and RCP components are identical, the phase difference between LCP and RCP in the outgoing light remains constant, and the polarization state remains unchanged in both the frequency and time domains (red star marker on the Poincaré sphere). When *α*=-45°, the polarization state on the Poincaré sphere in the frequency domain changes from x-polarized to nearly y-polarized in a clockwise direction, while in the time domain, the polarization state transitions from right-handed to left-handed. It is noteworthy that, unlike previously achieved time-varying polarization states, this method avoids the complex optical systems of Fourier synthesis.


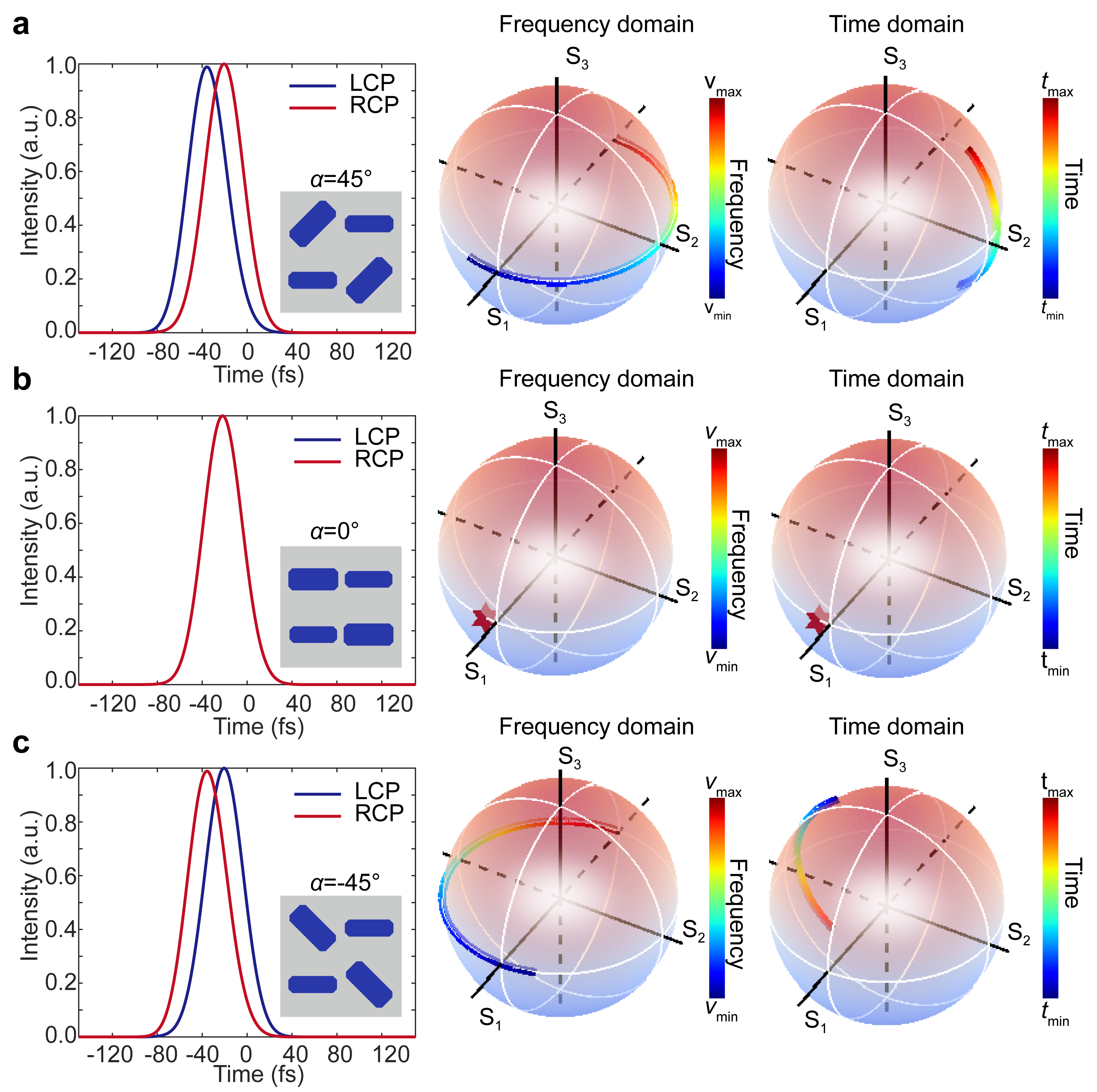


Fig.S11:Time-varying polarization state generation via the monolayer metasurface. Left panel (**a-c**). Temporal evolution of the intensities of the RCP and LCP components of the reflected light for three different supercells. An incident linearly polarized ultrafast pulse undergoes decomposition upon interaction with the metasurface, resulting in two split pulses. Middle panel (**a-c**): Evolution of the polarization state over time within the unitary structure. Right panel (**a-c**): Evolution of the polarization state over time within the unitary structure.

To generate a spatiotemporal vector optical field, the metasurface needs to apply different dispersion and phase to LCP and RCP. At the center frequency, RCP and LCP carry topological charges of +2 and -1, respectively. Additionally, both LCP and RCP have focus phases, as shown in Eq. S19, where *f* = 100 μm. We designed different spatially varying dispersions for LCP and RCP, as shown in Fig. 5.

We discretized the frequency range of 195 THz to 225 THz into *N* frequency points. We first used RCWA to calculate the optical responses of numerous supercell structures, with variables being the size and orientation angle of the subcells, and established a database. Under the aforementioned dispersion distribution, the ideal phase for each frequency is as follows:

Here, *C_L_* and *C_R_* are constants that remain unchanged with frequency to preserve phase coherence. The selection of supercells is then made using the following formula:

The vector angular spectrum theory was used to calculate the field distribution at the focal plane:

$$\left[\begin{matrix} E_{x}(x,y,\nu,z) \\ E_{y}(x,y,\nu,z) \\ E_{z}(x,y,\nu,z) \end{matrix}\right]=\iint\left[\begin{matrix} A_{x}(k_{x},k_{x}) \\ A_{y}(k_{x},k_{y}) \\ A_{z}(k_{x},k_{y}) \end{matrix}\right]e^{i(k_{x}x+k_{y}y+k_{z}z)}\text{d}k_{x}\text{d}k_{y}a$$

Here,$k_{z}=\sqrt{1-k_{x}^{2}+k_{y}^{2}}$, *A_z_*=-(*k_x_A_x_*+*k_y_A_y_*)/*k_z_*, We conducted vector diffraction calculations on each frequency component of LCP and RCP individually, yielding the electric fields *E_L_(x,y,z,ν)* and *E_R_(x,y,z,ν)* at the focal plane. Finally, the electric fields of all frequencies were summed to form the synthesized spatiotemporal vector optical field:

$$\begin{matrix} \left[\begin{matrix} E_{x}(x,y,z,t) \\ E_{y}(x,y,z,t) \end{matrix}\right]=\frac{\sqrt{2}}{2}E_{L}(x,y,z,t)\left[\begin{matrix} 1 \\ i \end{matrix}\right]+\frac{\sqrt{2}}{2}E_{R}(x,y,z,t)\left[\begin{matrix} 1 \\ -i \end{matrix}\right] \\ E_{L}(x,y,z,t)=\sum_{\nu} E_{L}(x,y,z,\nu)e^{i2\pi\nu t} \\ E_{R}(x,y,z,t)=\sum_{\nu} E_{R}(x,y,z,\nu)e^{i2\pi\nu t} \end{matrix}$$

where *E_L_* and *E_R_* represent the LCP and RCP components, respectively. (*x*, *y*, *z*, *t*) are the coordinate and time, respectively.

## S6. Fabrications


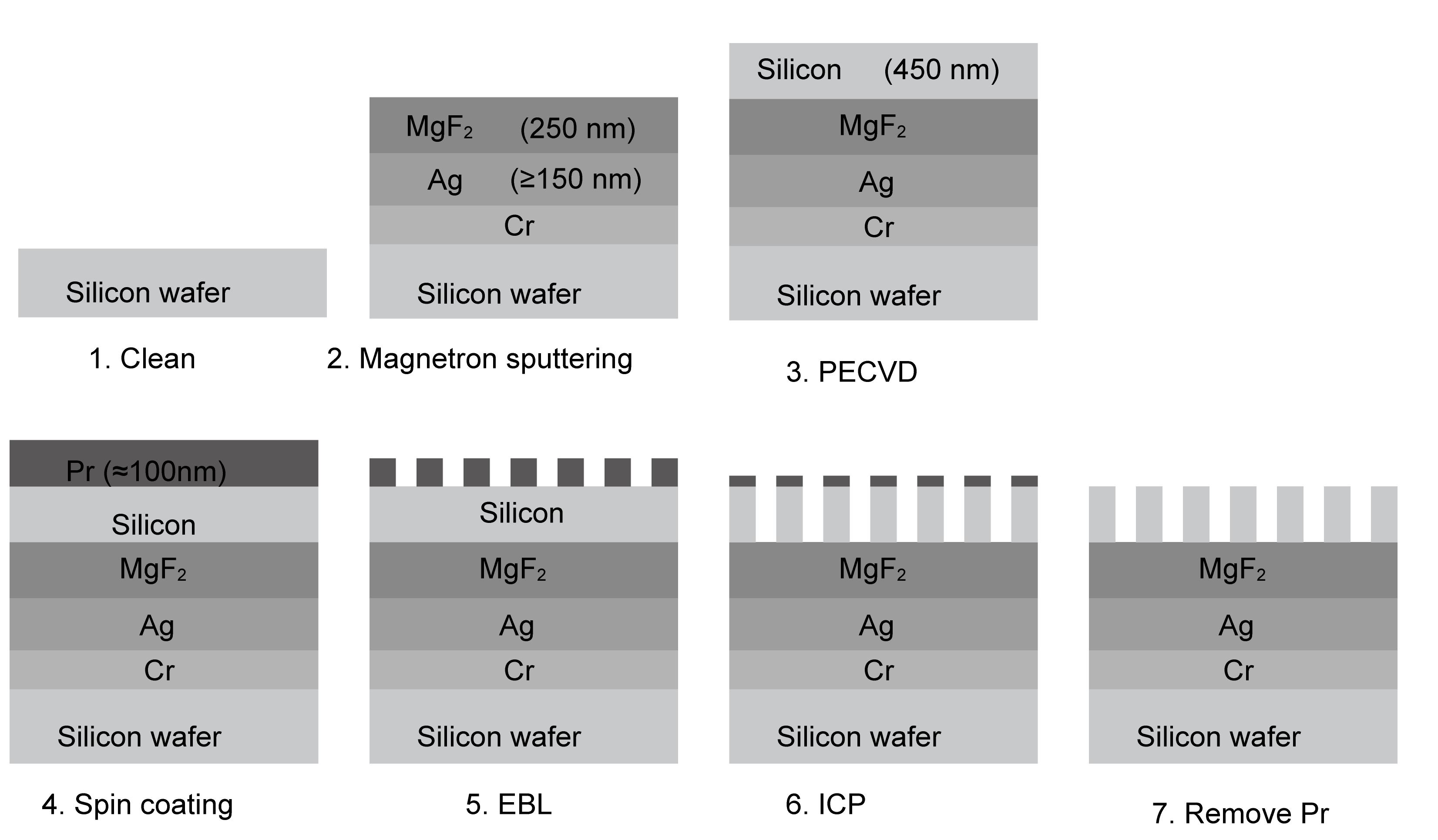


Fig.S12: Schematic diagram of the sample fabrication process. PECVD: plasma-enhanced chemical vapor deposition; Pr: photoresist, maN2401; EBL: electron beam lithography; ICP: inductively coupled plasma etching.

## S7. Characterizations


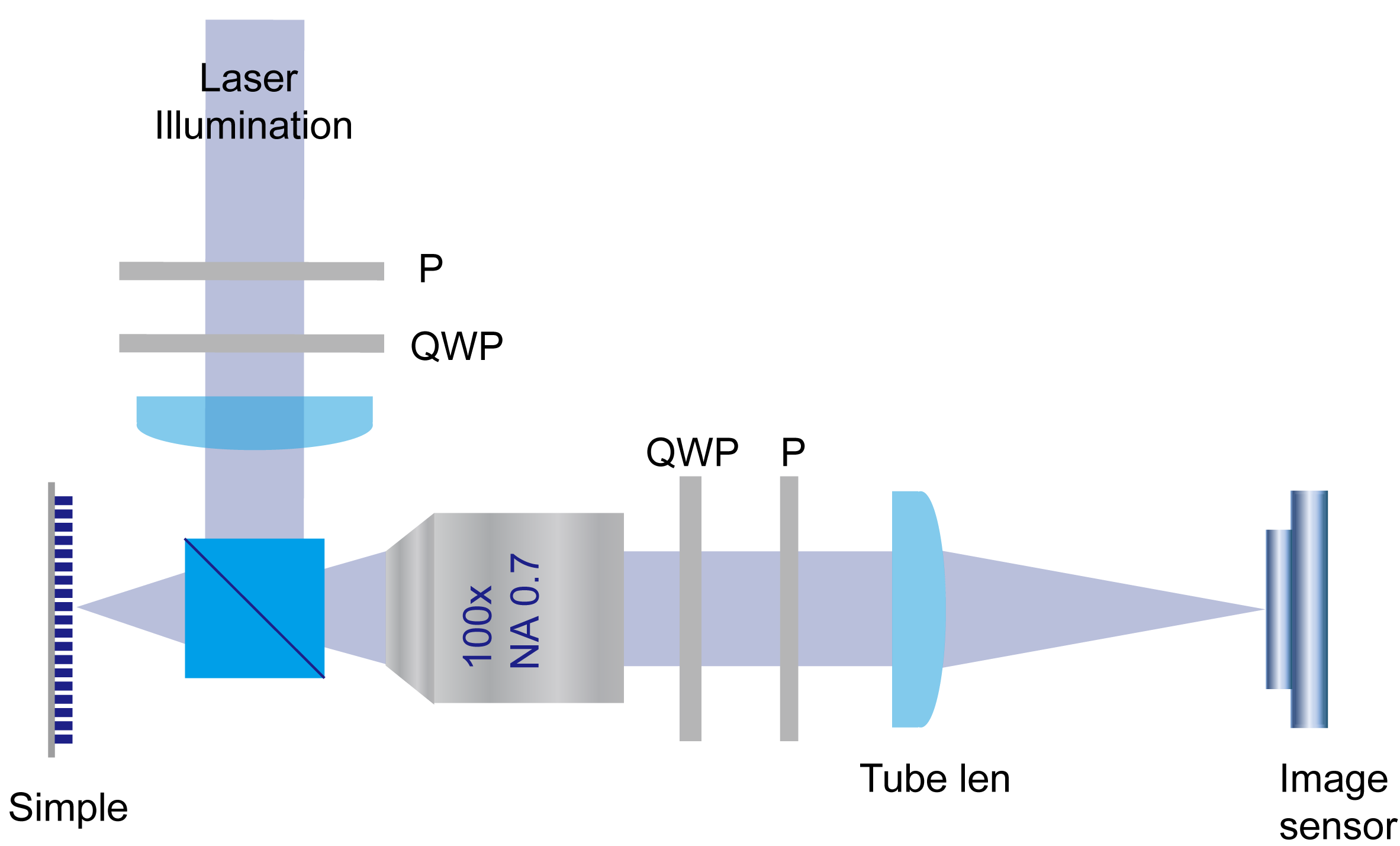


Fig.S13: Diagram of the optical setup for characterizations of the samples. P: linear polarizer; QWP: quarter waveplate.

## S8. Physics-data-driven optimization model

The meta-devices discussed in the main manuscript feature small phase and dispersion gradients, resulting in gradual changes in supercells and negligible crosstalk between adjacent supercells. Therefore, inverse design optimization is not necessary. When the phase and dispersion gradients are large or to further enhance the performance, we present a physics-data-driven optimization model that integrates adjoint shape optimization with deep learning. In the following, we provide a general outline of the technical approach and algorithmic steps. More details have been presented in our previous work.^66, 67^

As an example, consider the high-NA achromatic polarization-multiplexed metalens. The optimization model can be divided into two components: adjoint optimization, shown in Fig. S14a, and deep learning, shown in Fig. S14b. The details of the adjoint optimization have been thoroughly described in our previous work (Ref [66]), covering aspects such as the definition of the objective function, the form of the adjoint field, and the update procedure for the structure. Achromatization imposes a multi-objective optimization problem. The design wavelength range can be approximated by several discrete wavelengths *λ_k_*, and for each wavelength, the objective function is defined as follows.：

$$\text{FoM}_{R/L}(\lambda_{k})=|\langle\mathbf{E}_{R/L}^{\text{d}}(\lambda_{k})|\mathbf{E}_{R/L}^{\text{fwd}}(\lambda_{k})\rangle|$$

Here, $\text{E}_{R/L}^{\text{d}}$ refers to the ideal electric field for each wavelength, where the ideal field for the focusing metalens is the converging spherical wave. $\text{E}_{R/L}^{\text{fwd}}$ represents the reflected field resulting from the modulation of a plane wave incident on the metasurface. R and L denote right-handed circular polarization (RCP) and left-handed circular polarization (LCP), respectively. Forward simulations and adjoint simulations are conducted for each wavelength to obtain the structural deformation. The total structural deformation can be calculated using either the average value or by applying specific weights. Through several iterations, the optimization of the high-efficiency, high-NA achromatic metalens can be achieved. As full-wave simulations are performed for the entire metalens, the crosstalk between adjacent supercells is fully incorporated into the optimization process.


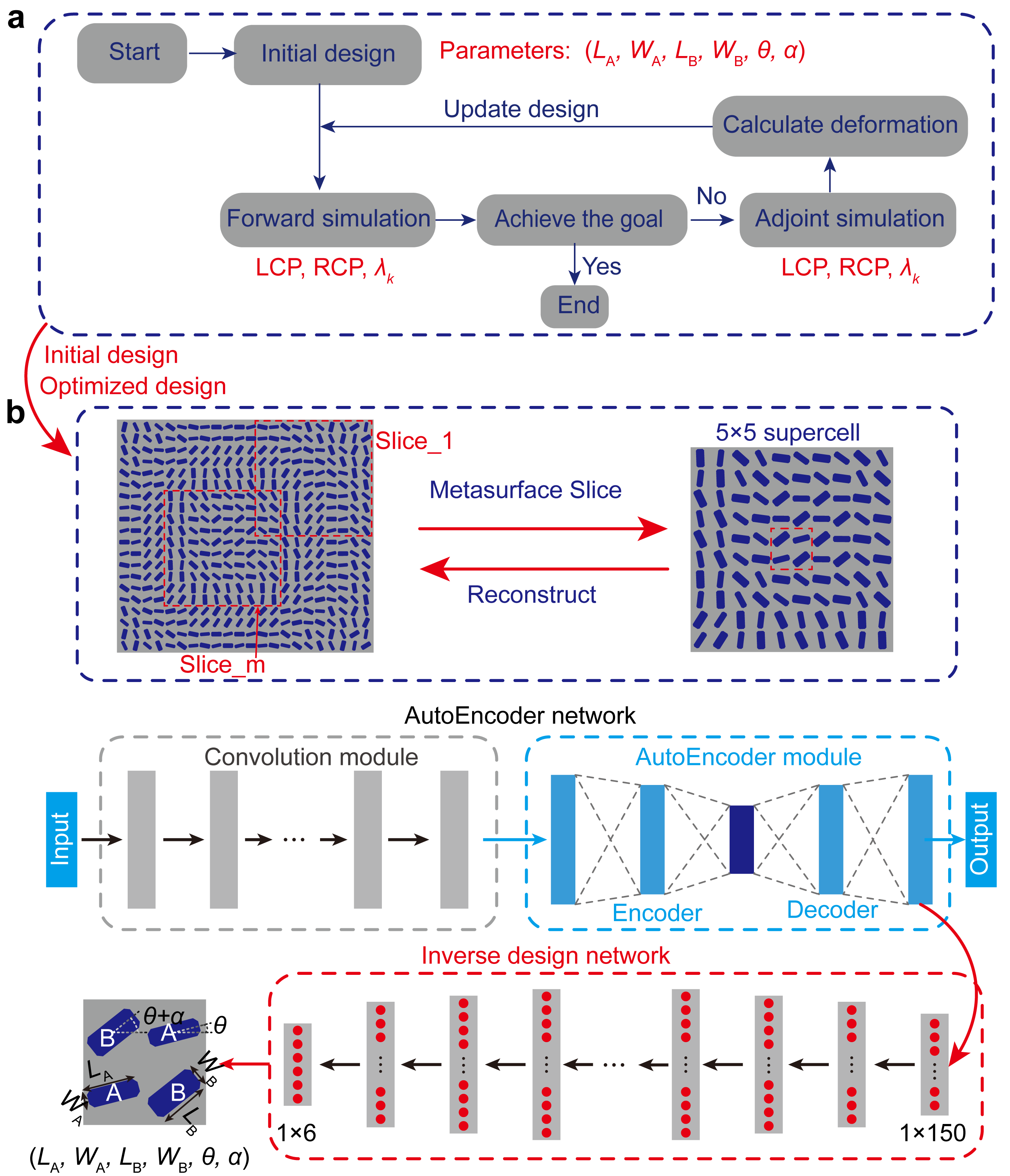


Fig.S14: Optimization model combining adjoint optimization and deep learning.

The adjoint optimization method shown in Fig. S14a is effective for optimizing small-sized metalenses (diameter < 100*λ*). For large-scale metasurfaces, a combination with deep-learning techniques, as illustrated in Fig. S14b, is required. The details of this approach are extensively described in our previous work (Ref [67]). In general, the metalens structure parameters P^b^ before the adjoint optimization are used as input data, and the optimized structure parameters P^a^ after the adjoint optimization are the output data, which are used to train the deep-learning model. Notably, the data utilized for training the deep-learning model is derived from small-scale metasurfaces. After the model is trained, the initial design parameters of the large metasurface are input, and the deep-learning model outputs the optimized structure parameters. Since crosstalk occurs between adjacent supercells, the model starts by slicing the metasurface into numerous 5×5 datasets with a slice interval of 1. Each dataset contains 25 supercells, and each supercell has 6 parameters. These parameters are input into the deep-learning model, which processes the data using the AutoEncoder network and the Inverse design network. The final output consists of the 6 structural parameters of the central supercell. In other words, the parameters of the central supercell are collectively determined by the surrounding supercells. Through repeated training iterations, the model parameters are adjusted to minimize the error between the predicted parameters P^o^ and the output data P^a^. When designing large-scale metasurfaces, the metasurface is similarly sliced into 5×5 datasets, which are processed by the trained deep-learning model to optimize the parameters for the central supercell. The final large-scale metasurface is then reconstructed by reassembling the datasets according to the original slicing order.
